# Loss of DDRGK1 impairs IRE1α UFMylation in spondyloepiphyseal dysplasia

**DOI:** 10.7150/ijbs.82765

**Published:** 2023-09-04

**Authors:** Xiao Yang, Tangjun Zhou, Xin Wang, Ying Xia, Xiankun Cao, Xiaofei Cheng, Yu Cao, Peixiang Ma, Hui Ma, An Qin, Jie Zhao

**Affiliations:** 1 Shanghai Key Laboratory of Orthopedic Implants, Department of Orthopedics, Ninth People's Hospital, Shanghai Jiao Tong University School of Medicine, Shanghai, China.; 2 Institute of Precision Medicine, The Ninth People's Hospital, Shanghai Jiao Tong University School of Medicine, Shanghai, China.

**Keywords:** spondyloepiphyseal dysplasia, DDRGK1, IRE1α, endoplasmic reticulum stress, apoptosis

## Abstract

Spondyloepiphyseal dysplasia (SEMD) is a rare disease in which cartilage growth is disrupted, and the DDRGK1 mutation is one of the causative genes. In our study, we established *Ddrgk1*^fl/fl^, *Col2a1*-ERT Cre mice, which showed a thickened hypertrophic zone (HZ) in the growth plate, simulating the previous reported SEMD pathology *in vivo*. Instead of the classical modulation mechanism towards SOX9, our further mechanism study found that DDRGK1 stabilizes the stress sensor endoplasmic reticulum-to-nucleus signaling 1 (IRE1α) to maintain endoplasmic reticulum (ER) homoeostasis. The loss of DDRGK1 decreased the UFMylation and subsequently led to increased ubiquitylation-mediated IRE1α degradation, causing ER dysfunction and activating the PERK/CHOP/Caspase3 apoptosis pathway. Further DDRGK1 K268R-mutant mice revealed the importance of K268 UFMylation site in IRE1α degradation and subsequent ER dysfunction*.* In conclusion, DDRGK1 stabilizes IRE1α to ameliorate ER stress and following apoptosis in chondrocytes, which finally promote the normal chondrogenesis.

## Introduction

Spondyloepiphyseal dysplasia (SEMD) is a genetic disease that is caused by mutations in multiple genes and results in impaired cartilage growth and development 1. The main SEMD characteristics in patients are short stature and early onset of joint and spinal degeneration 2-4. Among the various genes that have been reported to cause SEMD, a loss-of-function mutation (a homozygous c.408+1G>A donor splice site mutation) in the *DDRGK1* domain-containing (*DDRGK1*) gene is garnering interest in this research field 5, 6.

DDRGK1, also known as ubiquitin-fold modifier 1 protein (UFM1)-binding protein 1 with proteasome, COP9, and initiation factor 3 domain (UFBP1), is a key member of the ubiquitin-like protein family 7, 8. Thus, DDRGK1 and other proteins, such as UFL1, UBA5 and UFC1, are intricately involved in a process called UFMylation, in which proteins are tagged with a UFM1 group 9, 10. Deletion of the DDRGK1 gene has been demonstrated to inhibit the phosphorylation of an inhibitor of the NF-κB α subunit to protect it from ubiquitylation-mediated degradation in U2OS cells 11. In addition, the DDRGK1 protein has been shown to be involved in the activation of estrogen receptor α by regulating the activation of its downstream component activating signal cointegrator 1 (ASC1) and promoting its UFMylation in MCF-7 cells 12. Importantly, Adetutu *et al* previously reported that the loss of DDRGK1 in zebrafish led to the reduced expression of the cartilage-specific protein SRY-box transcription factor 9 (SOX9), causing chondrogenic disorders 5. Furthermore, this group suggested that the same mechanism involved in the loss of DDRGK1 impaired the expression of SOX9 and chondrogenesis in growth plate cells of *Ddrgk1*^fl/fl^, *Prx.1* Cre and *Ddrgk1*^fl/fl^, *Aggrecan*-ERT Cre mice, causing an abnormally increased HZ 13.

The DDRGK1 protein is located at the endoplasmic reticulum (ER) membrane; however, studies have primarily focused on the expression of proteins related to growth, development and transcription, and little attention has been directed to study the relationship between ER stress pathways and DDRGK1 action during cell differentiation. Recently, a study reported that DDRGK1 maintained the stability of IRE1α 7, and increasing evidence has shown that decreased DDRGK1-induced ER stress is associated with a variety of diseases, including cancer, neurodegeneration, diabetes, and proinflammatory diseases 14-18. In plasma cells, the loss of DDRGK1 blocked ER expansion and immunoglobulin production 19. In tumor cells, including breast and liver cancer cells, DDRGK1 knockout was shown to induce ER stress and promote apoptosis 20, and an imbalance of ER homeostasis led to altered chondrocyte differentiation in Col10a1 (13del) mice 21. Although the initial stages of the unfolded protein response (UPR) are cytoprotective mechanisms that restore ER homeostasis, chronic activation of the UPR can result in apoptosis 22, 23. These studies strongly suggested that decreased DDRGK1-induced ER stress might be one of the mechanisms causing impaired chondrogenesis in the growth plate cells of *Ddrgk1*-conditional knockout (cKO) mice 13. Therefore, in this study, we aimed to elucidate the contribution of DDRGK1-mediated ER stress to SEMD.

## Results

### Conditional knockout of DDRGK1 in *Ddrgk1*^fl/fl^, *Col2a1*-ERT Cre mice simulates the SEMD phenotype *in vivo*

**Figure 1:**
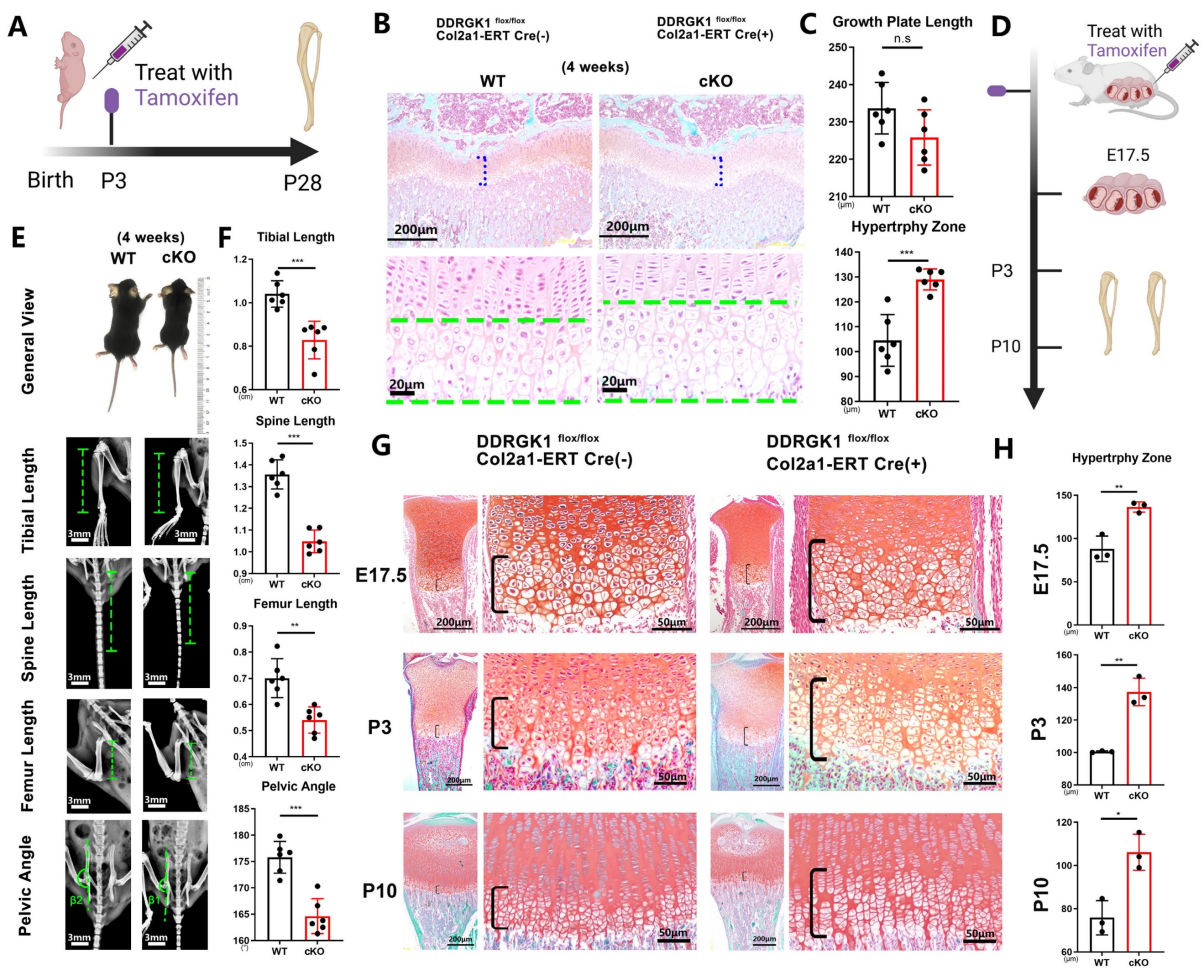
** Conditional knockout of DDRGK1 in *Ddrgk1*^fl/fl^, *Col2a1*-ERT Cre mice leads to the acquisition of the SEMD phenotype *in vivo.* (A)** Schematic showing tamoxifen-induced *Ddrgk1*-cKO mice sacrifice on P28. **(B)** Safranin O-Fast Green staining of the lower limbs of the WT and cKO mice is shown; the growth plate is the focus. **(C)** Quantification of the length of the growth plate and hypertrophy zone (HZ) shown in panel (B). **(D)** Schematic showing tamoxifen-induced *Ddrgk1*-cKO mice sacrificed on E17.5, P3 or P10. **(E)** Photograph and radiograph test of the WT and cKO mice shown in panel (A). **(F)** Quantification of the tibial length, spine length, femur length and pelvic angle of the WT and cKO mice shown in panel (E). **(G)** Safranin O-Fast Green staining of the lower limbs of the WT and cKO mice shown in d; the growth plate is the focus. **(H)** Quantification of the length of the HZ shown in panel (G). All data are presented as the mean ± SD from three or more experiments. ^*^P < 0.05, ^**^P < 0.01, ^***^P < 0.001 and ^****^P < 0.0001.

**Figure 2:**
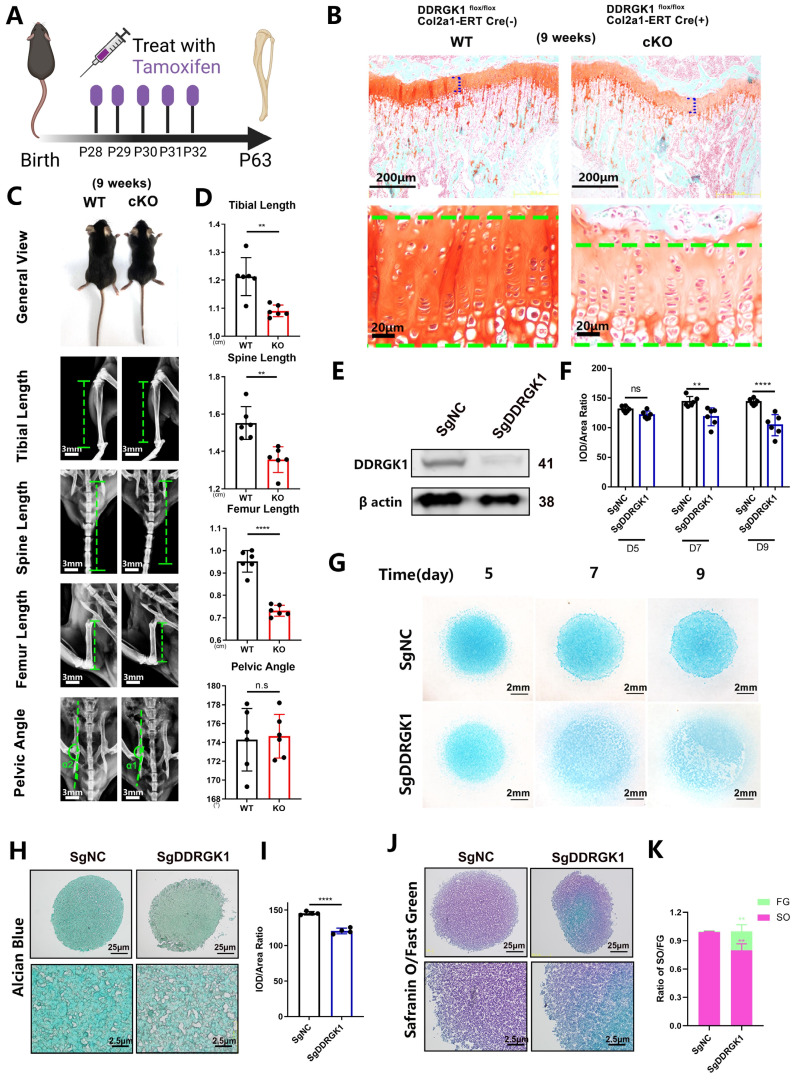
**Loss of DDRGK1 impaired chondrogenesis in growth plate cells *in vivo* and ATDC5 cells *in vitro.* (A)** Schematic showing tamoxifen-induced *Ddrgk1*-cKO mice sacrificed on P63. **(B)** Safranin O-Fast Green staining of the lower limbs in WT and cKO mice is shown; the growth plate is the focus. **(C)** Photograph and radiograph test of the WT and cKO mice shown in panel (A). **(D)** Quantification of the tibial length, spine length, femur length and pelvic angle of the WT and cKO mice shown in panel (C). **(E)** Western blot analysis of the DDRGK1 protein in NC and KO ATDC5 chondrocytes using β-actin as a reference. **(F)** Quantification of the integrated optical density (IOD)/area ratio of the Alcian blue-stained cells shown in panel (G). **(G)** Alcian blue staining of NC and KO ATDC5 chondrocytes 5, 7 and 9 days after high-density culturing in chondrogenesis medium. **(H)** Alcian blue staining of NC and KO ATDC5 chondrocytes 21 days after pellet culturing in chondrogenesis medium. **(I)** Quantification of the IOD/area ratio of the Alcian blue-stained cells shown in panel (H).** (J)** Safranin O-Fast Green staining of NC and KO ATDC5 chondrocytes 21 days after pellet culturing in chondrogenesis medium.** (K)** Quantification of the Safranin O (SO)/Fast Green (FG) ratio of the cells shown in panel (J). All data are presented as the mean ± SD from three or more experiments. ^*^P < 0.05, ^**^P < 0.01, ^***^P < 0.001 and ^****^P < 0.0001.

Three days after birth, DDRGK1 was conditionally knocked out in *Ddrgk1*^fl/fl^, *Col2a1*-ERT Cre mice using tamoxifen (TMX) (**Figure 1A**). Then, on postnatal day 28, the X-ray images revealed that the cKO mice presented a SEMD phenotype (**Figure 1E**). The tibial length, spine length, and femur length were significantly shorter, with pelvic angle significantly smaller in the cKO mice than in the WT mice (**Figure 1F**). Safranin O-Fast Green staining showed that the length of the HZ in the cKO mice was longer than that in the WT mice (**Figure 1B, C**). Moreover, the cKO mice also presented with an increased HZ in the cartilage end plate of the intervertebral disc (**Supplementary Figure 1a**). To further understand the imbalanced differentiation process in the WT and cKO mice, we knocked out DDRGK1 with pre-natal induction (TMX) and found that the growth curves of mass and length were significantly retarded in cKO mice (**Supplementary Figure 2a, b**). At three different time points: embryonic day (E)17.5, postnatal day (P) 3 and P10 (**Figure 1D**), the loss of DDRGK1 significantly impaired the normal differentiation of chondrocytes in the growth plates. The thickness of the hypertrophy zone in the cKO mice was greater than that in WT mice (**Figure 1G, H**). In the 9-week-old cKO mice in which DDRGK1 was knocked out from P28 to P32 (**Figure 2A**), the thickness of the growth plate in cKO mice was significantly shorter (**Figure 2B and Supplementary Figure 3b**). Moreover, we observed the same SEMD phenotype in the radiographs of the cKO mice (**Figure 2C**), which presented with a decrease in tibial length, spine length and femur length (**Figure 2D**) but not in pelvic angle. The spine in the cKO mice also followed the same trend (**Supplementary Figure 3a**). Moreover, there is no gender difference in the SEMD phenotype that we found that the female cKO mice also showed decreased tibial length, spine length, and femur length (**Supplementary Figure 2c, d**). These *in vivo* results indicated that DDRGK1 plays a role in chondrocyte differentiation in the growth plate.

### Loss of DDRGK1 impaired chondrogenesis via apoptosis and ER stress signaling pathways

To study the underlying mechanism of DDRGK1 in chondrogenesis, we established a SgDDRGK1 knockout ATDC5 cell line (**Figure 2E**) and evaluated chondrogenesis in a high-density culture system. *Ddrgk1* knockout (KO) cells showed markedly less Alcian blue staining than negative control (NC) cells (**Figure 2F, G**). Using a pellet culture system, we also observed that KO cells exhibited markedly less Alcian blue staining than NC cells, and the difference was particularly striking in the central area of the cell pellet (**Figure 2H, I**). According to Safranin O-Fast Green staining assays, the majority of the NC cell pellet was stained with Safranin O, but the central part of the KO cell pellet was stained only with Fast Green, suggesting that chondrogenesis had been disrupted (**Figure 2J, K**). Moreover, the expression of important chondrocyte markers, including aggrecan 24 and SOX9 25 decreased in KO cells (**Supplementary Figure 4a, b and c**).

**Figure 3:**
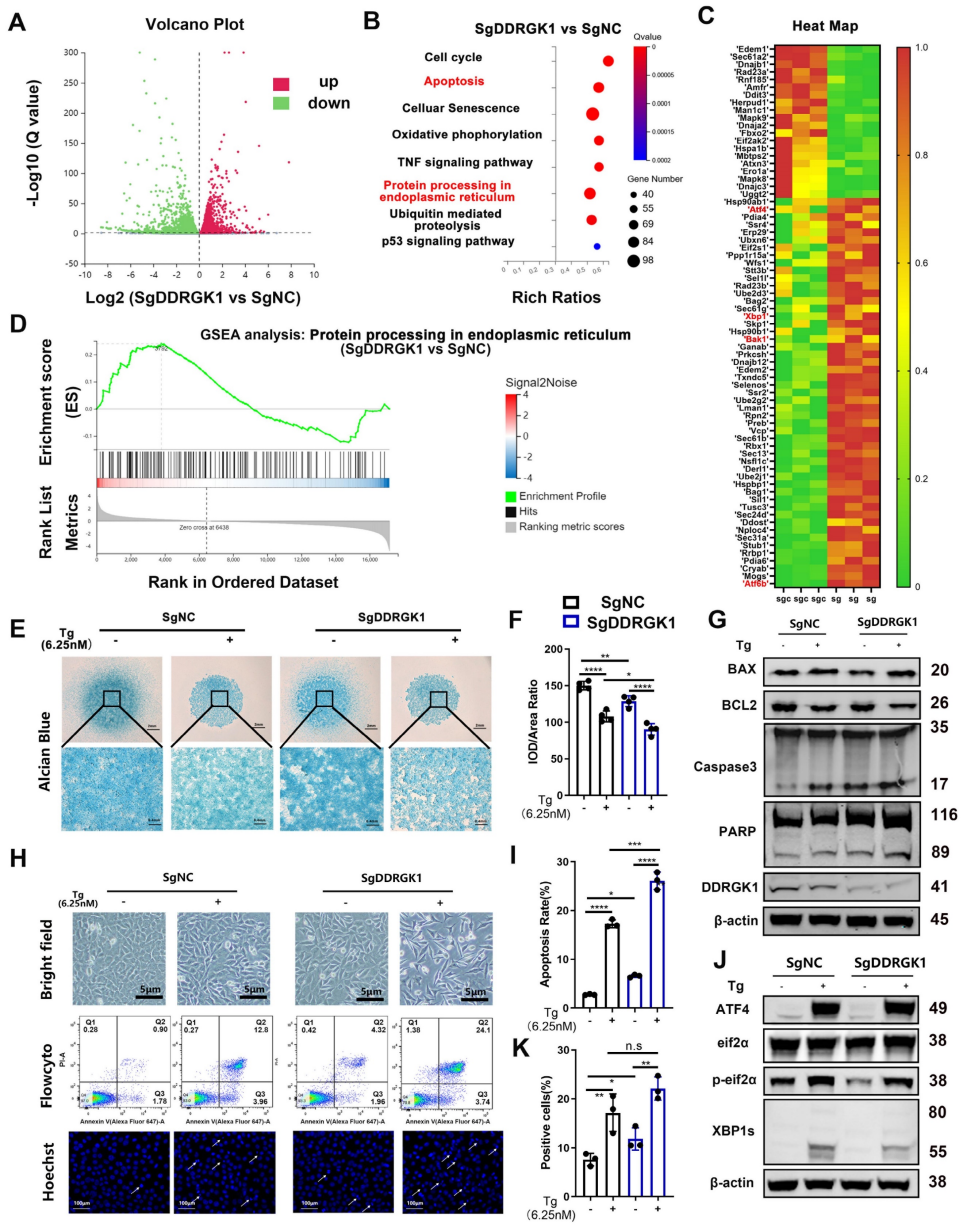
** Loss of DDRGK1 in ATDC5 cells led to impaired chondrogenesis with apoptosis and ER stress signaling pathway involvement. (A)** Ratio of mRNA level change in NC and KO ATDC5 chondrocytes in a volcano plot (3 pairs of biological replicates). **(B)** KEGG pathway analyses of differentially expressed mRNAs in the NC and KO ATDC5 chondrocytes shown in panel (A). **(C)** Heatmap showing the mRNAs of interest in the NC and KO ATDC5 chondrocytes shown in panel (A). **(D)** Gene set enrichment analysis (GSEA) of ER stress pathways in the NC and KO ATDC5 chondrocytes shown in panel (A). **(E)** Alcian blue staining of NC and KO ATDC5 chondrocytes after 9 days of high-density culture in chondrogenesis medium with or without *thapsigargin* (Tg) (6.25 nM).** (F)** Quantification of the integrated optical density /area ratio in the Alcian blue-stained cells shown in panel (E). Cell morphology of NC and KO ATDC5 chondrocytes with or without Tg (6.25 nM) treatment. **(G)** Western blot analysis of Bax, Bcl2, cleaved and full-length Caspase 3, cleaved and full-length PARP, DDRGK1 and β-actin expression in NC and KO ATDC5 chondrocytes with or without Tg (6.25 nM) treatment. **(H)** Cell morphology, flow cytometry and Hoechst staining of NC and KO ATDC5 chondrocytes treated with or without Tg (6.25 nM) for 24 h. Karyopyknosis of the cell nucleus was observed using Hoechst staining. **(I)** Quantification of the apoptosis rate (Q2 + Q3) based on flow cytometry of the cells shown in panel (H). **(J)** Western blot analysis of ATF-4, eIF2α, phospho-eIF2α and XBP-1s control in NC and KO ATDC5 chondrocytes treated with Tg for 24 h using β-actin as the loading control. **(K)** Quantification of karyopyknosis cells based on the number of cells with shrunken nuclei and the total number of cells with normal nuclei shown in panel (H) after Hoechst staining. All data are presented as the mean ± SD from three or more experiments. ^*^P < 0.05, ^**^P < 0.01, ^***^P < 0.001 and ^****^P < 0.0001.

The mechanism disrupting chondrogenesis was analyzed via RNA-seq assay. The differentially expressed genes between the KO and NC cells were identified in a volcano plot (**Figure 3A**). A KEGG pathway analysis showed that apoptosis, cell cycle, cell senescence and P53 signaling pathways were mainly involved. As DDRGK1 is located at the ER membrane, the ER stress signaling pathway was also involved (**Figure 3B**). Among the differentially expressed genes, the levels of the apoptosis marker Bak1 and the ER stress markers ATF4, XBP1sand ATF6b increased when loss of DDRGK1 (**Figure 3C**). A gene set enrichment analysis (GSEA) focused on “protein processing in endoplasmic reticulum” also indicated that these ER stress genes participated in chondrogenesis (**Figure 3D**). Moreover, a GSEA focused on “apoptosis” revealed that the apoptosis pathway might be particularly important in dysregulated chondrogenesis (**Supplementary Figure 3c**).

### Loss of DDRGK1 led to apoptosis, especially in conjunction with ER stress

Considering the possibility that ER stress and apoptosis pathways are highly involved in the chondrogenesis process, we used *thapsigargin* (Tg) as the ER stress trigger for further studies. The extracellular matrix (ECM) in NC cells was degraded by Tg treatment, and KO further deteriorated the ECM degradation (**Figure 3E, F**). The viability of KO cells was found to be significantly reduced compared with that of NC cells (**Supplementary Figure 4d**). In addition, Tg profoundly reduced the viability of both NC and KO cell lines (**Supplementary Figure 4d**). Loss of DDRGK1 caused cell swelling, and following treatment with Tg, the KO cells exhibited additional features of apoptosis (**Figure 3H**). In addition, more KO underwent apoptosis, and after Tg treatment, the apoptosis rate was higher in the KO cells than in the NC cells (**Figure 3H, I**). Hoechst staining showed that the cells appeared to be stretched with significantly increased nuclear pyknosis (**Figure 3H, K**). By performing a Western blot analysis, we found increased Bax expression and decreased Bcl-2 expression in both NC and KO cells after Tg treatment (**Figure 3G and Supplementary Figure 4e**). Furthermore, the Bcl-2/Bax ratio was significantly decreased in the KO cells compared with that in the NC cells after Tg treatment (**Supplementary Figure 4e**). The protein levels of the end-stage apoptosis markers cleaved Caspase 3 and cleaved PARP were also increased in the KO cells, and these levels were increased after Tg treatment (**Figure 3G**). However, no changes in the expression of the pyroptosis marker GSDMD or the necrosis marker RIP was observed after Tg treatment (**Supplementary Figure 4f**), suggesting that the loss of DDRGK1 led to cell death was mediated through the Bax/Bcl-2/Caspase 3 apoptosis signaling pathway, not the pyroptosis or necrosis pathways. To further prove the apoptosis and ER stress pathways, we isolated the primary chondrocytes from WT and cKO mice induced with TMX (20μM) (**Supplementary Figure 5a**). Loss of DDRGK1 in primary chondrocytes aggravated the apoptosis in Flowcyto test (**Supplementary Figure 5a, b**) and the protein expression of cleaved PARP and Caspase-3 increased in KO chondrocytes (**Supplementary Figure 5c**). The inner mechanism also might be the deterioration of ER stress in KO chondrocytes, we could observe the decreased IRE1α, XBP-1s, and increased BIP, ATF4, and CHOP expression in WB test (**Supplementary Figure 5d**). Following with this, the differentiation of primary chondrocytes in cKO mice was also blocked (**Supplementary Figure 5e, f**). With respect to the ER pathways, we found that the protein expression of XBP1s decreased and that of ATF4 did not change in the KO cells (**Figure 3J**), which differed from the RNA-seq results (**Figure 3C**), meaning a posttranslational mechanism may be involved in the regulatory process.

### DDRGK1 interacts with IRE1α at multiple sites

**Figure 4:**
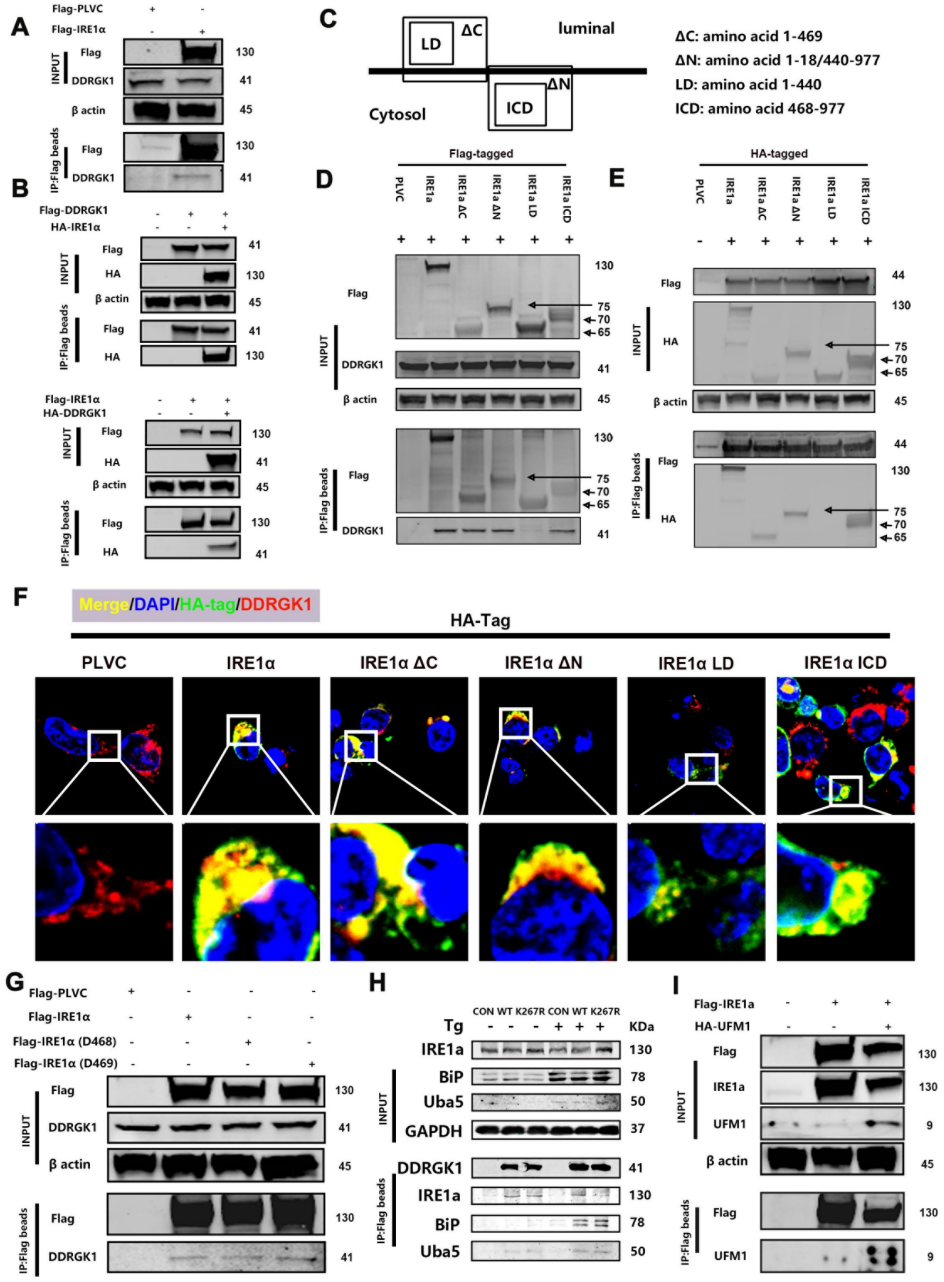
**DDRGK1 interacts with IRE1α at multiple sites *in vitro***.** (A)** Coimmunoprecipitation analysis performed to determine the possible interaction between Flag-IRE1α and DDRGK1 using Flag-tagged beads with 293T cells. **(B)** Coimmunoprecipitation analysis performed to determine the possible interaction between Flag-DDRGK1 and HA-IRE1α and between Flag-IRE1α and HA-DDRGK1 using Flag-tagged beads with 293T cells. **(C)** Graphical illustration of HA (Flag)-IRE1α ΔC, HA (Flag)-IRE1α ΔN, HA (Flag)-IRE1α LD and HA (Flag)-IRE1α ICD. **(D)** Coimmunoprecipitation analysis performed to determine the possible interaction between Flag-IRE1α, Flag-IRE1α ΔC, Flag-IRE1α ΔN, Flag-IRE1α LD or Flag-IRE1α ICD and DDRGK1 using Flag-tagged beads with 293T cells. **(E)** Coimmunoprecipitation analysis performed to determine the possible interaction between Flag-DDRGK1 and HA-IRE1α, HA-IRE1α ΔC, HA-IRE1α ΔN, HA-IRE1α LD or HA-IRE1α ICD using Flag-tagged beads in 293T cells. **(F)** Immunofluorescence colocalization analysis showing HA-PLVC, HA-IRE1α, HA-IRE1α ΔC, HA-IRE1α ΔN, HA-IRE1α LD, and HA-IRE1α ICD with DDRGK1 in 293T cells. **(G)** Coimmunoprecipitation analysis performed to determine the possible interaction between Flag-IRE1α, Flag-IRE1α D468 or Flag-IRE1α D469 and intrinsic DDRGK1 using Flag-tagged beads with 293T cells. **(H)** Coimmunoprecipitation analysis performed to determine the possible interaction between Flag-DDRGK1 or the Flag-DDRGK1 K267R mutant and intrinsic IRE1α, BIP or UBA5 using Flag-tagged beads with 293T cells with or without *thapsigargin* (Tg) treatment for 24 h.** (I)** Coimmunoprecipitation analysis to determine the possible interaction between Flag-IRE1α and HA-UFM1 using Flag-tagged beads with 293T cells.

The differentially expressed protein XBP1s is a protein downstream from IRE1α, and DDRGK1 is localized to the ER and interacts with IRE1α 26, thereby regulating ER stress partially through the IRE1α/ER-associated degradation (ERAD) pathway 27. Next, we focused on the DDRGK1 and IRE1α relationship, especially their interaction sites. We transfected 293T cells with Flag-IRE1α to pull down any interacting protein partners, and DDRGK1 was identified (**Figure 4A**). Co-transfecting cells with either Flag-DDRGK1 and hemagglutinin (HA)-IRE1α or with Flag-IRE1α and HA-DDRGK1 confirmed the interaction between DDRGK1 and IRE1α (**Figure 4B**). Then, we constructed the following four plasmids encoding truncated versions of IRE1α for transfection: the transmembrane sequence named ΔC, which spanned amino acid residues 1-469; the transmembrane sequenced named ΔN, in which amino acid residues 19-439 were deleted; the ER luminal domain named LD, which consisted of amino acids 1-440; and the cytosol domain named ICD, which consisted of amino acids 468-977 (**Figure 4C**). After cotransfecting these constructs into 293T cells with Flag-DDRGK1, we found that DDRGK1 interacted with IRE1α at multiple sites; that is, we identified an interaction between Flag-DDRGK1 and IRE1α ΔC, IRE1α ΔN, and IRE1α ICD but not between Flag-DDRGK1 and IRE1α LD (**Figure 4D, E and F**). Notably, IRE1α ΔC and IRE1α ICD variants shared two amino acids, namely, residues 468 and 469. Therefore, we constructed plasmids encoding the Del468 (residue 468 deletion) and Del469 (residue 469 deletion) IRE1α variants. Neither of these two amino acids were found to be essential for the interaction between DDRGK1 and IRE1α, since deletion of either amino acid did not disrupt this interaction (**Figure 4G**). Regarding the interaction sites in DDRGK1, previous studies had confirmed that the lysine residue at the 267 site is the major site in this interaction 26 and that interactions at this site facilitated UFMylation 8, 12. We also confirmed this finding by showing that the K267R mutation in DDRGK1 abrogated the interaction between DDRGK1 and IRE1α, especially in the presence of Tg; however, this mutation did not affect the interaction between DDRGK1 and BIP (**Figure 4H**). We next transfected 293T cells with Flag-IRE1α or HA-UFM1, and we then identified an interaction between IRE1α and UFM1 (**Supplementary Figure 6a**). In addition to intrinsic interactions, in cells transfected with both plasmids, UFM1/IRE1α complexes were derived from exogenous Flag-tagged beads (**Figure 4I**).

### Loss of DDRGK1 led to impaired UFMylation and increased ubiquitination of IRE1α, which aggravated ER stress *in vitro*

**Figure 5:**
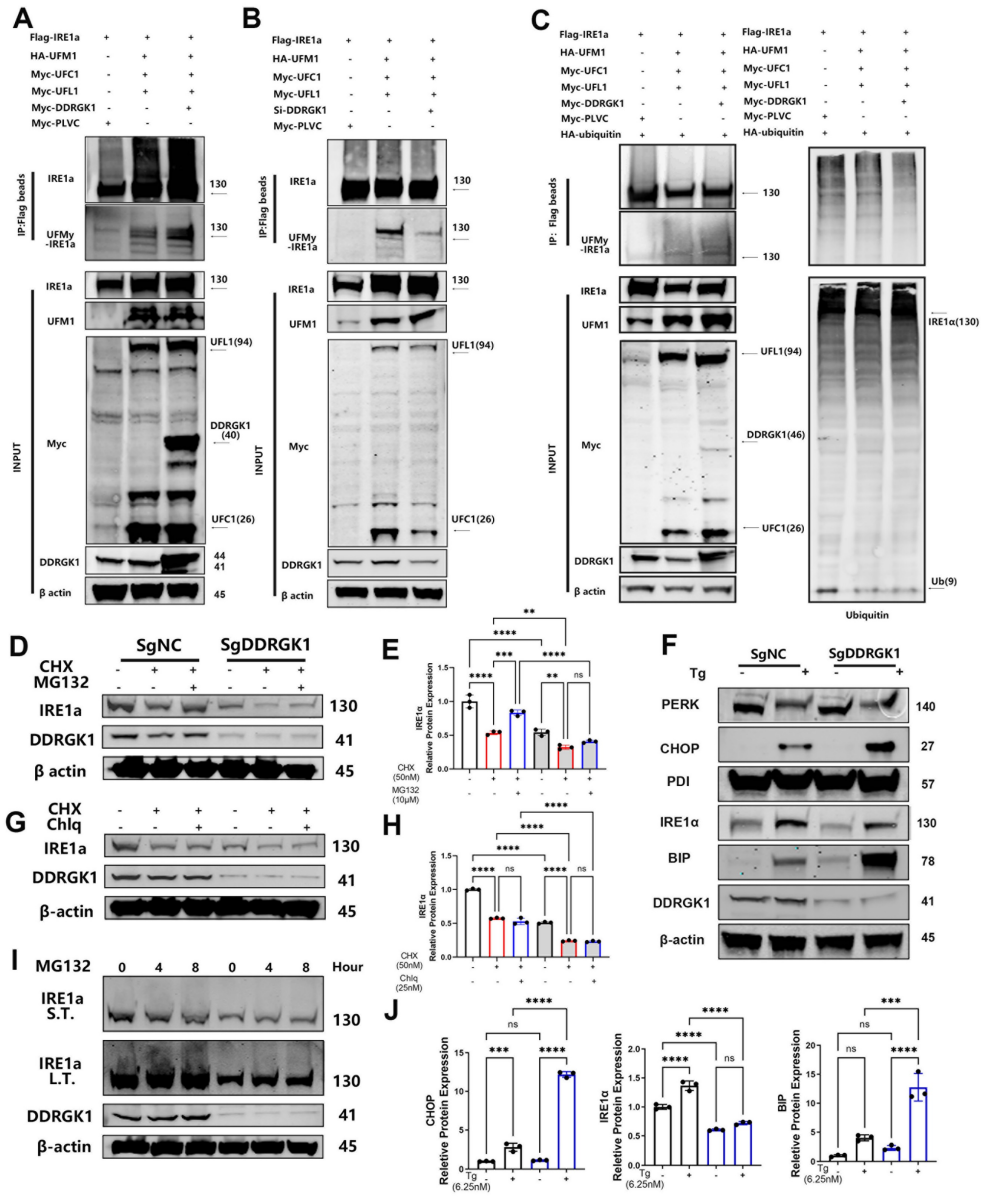
** Loss of DDRGK1 led to impaired UFMylation and increased ubiquitination of IRE1α, which aggravated ER stress *in vitro.* (A)** UFMylation analysis performed via pull-down assay of Flag-IRE1α with Flag-tagged beads after the Flag-PLVC, HA-UFM1, Myc-DDRGK1, Myc-UFL1 and Myc-UFC1 plasmid transfection of 293T cells. **(B)** UFMylation analysis performed via pull-down assay of Flag-IRE1α with Flag-tagged beads after Flag-PLVC, HA-UFM1, si-DDRGK1, Myc-UFL1 and Myc-UFC1 plasmid transfection of 293T cells. **(C)** UFMylation and ubiquitylation analysis of Flag-IRE1α after Flag-PLVC, HA-UFM1, Myc-DDRGK1, Myc-UFL1, Myc-UFC1 and HA-ubiquitin plasmid transfection of 293T cells. **(D)** Western blot analysis of IRE1α and DDRGK1 in NC and KO ATDC5 chondrocytes treated with cycloheximide (50 nM) and MG132 (10 μM); β-actin was the loading control. **(E)** Quantification of the gray values of IRE1α in the NC and KO ATDC5 chondrocytes shown in panel (D). **(F)** Western blot analysis of PERK, CHOP, PDI, IRE1α, BIP and DDRGK1 in NC and KO ATDC5 chondrocytes treated with thapsigargin (Tg) for 24 h; β-actin was the loading control. **(G)** Western blot analysis of IRE1α and DDRGK1 in NC and KO ATDC5 chondrocytes treated with cycloheximide (50 nM) and chloroquine (25 nM); β-actin was the loading control **(H)** Quantification of the gray values of IRE1α in the NC and KO ATDC5 chondrocytes shown in panel (G). **(I)** Western blot analysis of IRE1α and DDRGK1 in NC and KO ATDC5 chondrocytes treated with MG132 (10 μM) for 0, 4 and 8 h; β-actin was the loading control. L.T., long exposure time, and S.T., short exposure time. **(J)** Quantification of the gray values of IRE1α, CHOP and BIP in the NC and KO ATDC5 chondrocytes shown in panel (F). All data are < 0.001 and ^****^P < 0.0001.

To further decipher the function of DDRGK1 when bound to either IRE1α or UFM1, we established a poly-UFMylation system. The presence of DDRGK1 was found to enhance IRE1α UFMylation, whereas DDRGK1 knockdown impaired IRE1α UFMylation (**Figure 5A, B**). The increase in UFMylation led to the suppression of ubiquitination 28. In a ubiquitination assay, we co-transfected 293T cells with HA-IRE1α and Flag-ubiquitin, and then, we treated these cells with MG-132 for 12 h. The results showed that IRE1α was pulled down from samples incubated with HA-tagged beads, and polyubiquitylated bands were obtained (**Supplementary Figure 6b**). Then, we transfected Flag-ubiquitin alone into 293T cells. We successfully pulled down IRE1α using Flag-tagged beads (**Supplementary Figure 6d**). We observed that ubiquitylation of IRE1α was decreased while the levels of UFMylated IRE1α were increased after Myc-DDRGK1 was overexpressed (**Figure 5C**).

Imbalanced UFMylation and ubiquitination clearly influenced the protein expression of IRE1α in KO and NC ATDC5 cells, and the decrease in IRE1α mRNA expression was less dramatic in the KO cells following DDRGK1 knockout (**Supplementary Figure 7a, b**). We transfected these cell lines with Flag-IRE1α or HA-UFM1. Flag-tagged beads pulled down more quantities of UFM1 from the NC cells than from the KO cells (**Supplementary Figure 6c**). In contrast, HA-tagged beads failed to pull down IRE1α from the KO ATDC5 cells (**Supplementary Figure 6c**). The impaired UFMylation of IRE1α in the KO ATDC5 cells led to decreased IRE1α expression. After treating the cells with cycloheximide (CHX) to inhibit the synthesis of proteins, IRE1α expression decreased in both the KO and NC cells, and IRE1α expression was significantly reduced, falling to a negligible level in the KO cells (**Figure 5D, E**). In contrast, cotreating cells with MG-132 and CHX to inhibit proteasome activity resulted in the partial rescue of IRE1α expression in both the KO and NC cells (**Figure 5D, E**). In contrast to treatment with MG132, cotreatment of cells with CHX and chloroquine (Chlq), a lysosome and an autophagy inhibitor, respectively, did not restore IRE1α expression in either the KO or NC cells (**Figure 5G, H**). These outcomes suggested that the degradation of IRE1α in KO cells was mediated through the ubiquitin-associated proteasome degradation pathway not the lysosome-autophagy system. Subsequently, treatment of ATDC5 cells with MG-132 alone for 0, 4 or 8 h resulted in the time-dependent polyubiquitylation of IRE1α (**Figure 5I**). In addition, we observed “upshifting” of the bands, and the magnitude of this shift was greater in the NC cells than in the KO cells (**Figure 5I**).

IRE1α is one of the three transmembrane proteins in the ER membrane that can regulate ER stress in cells 29. Degradation of IRE1α leads to increased expression of the chaperone protein BIP. (**Figure 5F, J and Supplementary Figure 7c**). Using Tg as the ER stress trigger, we observed that IRE1α expression was increased (**Figure 5F, J and Supplementary Figure 7b**), with increased production of BIP; moreover, the increase in the level of BIP was more profound in the KO cells than in the NC cells (**Figure 5F, J and Supplementary Figure 7c**). The excessive accumulation of unfolded proteins activates another UPR pathway: the PERK/C/EBP homologous protein (CHOP) pathway. Activated PERK increases the rate of CHOP translocation into the nucleus (**Figure 5F, J and Supplementary Figure 7d**), which induces ER stress-mediated apoptosis. The mRNA expression levels of Bax were increased in KO cells compared with NC cells. This outcome coincided with a decrease expression in Bcl-2, chondrogenic marker Col2a1 and SOX9 in KO cells, which was further decreased after Tg treatment (**Supplementary Figure 7e, f, g, h**).

### Loss of DDRGK1 or the K268R mutation led to IRE1α degradation and aggravated ER stress in growth plate cells of mice* in vivo* and ATDC5 cells/primary chondrocytes* in vitro*

**Figure 6:**
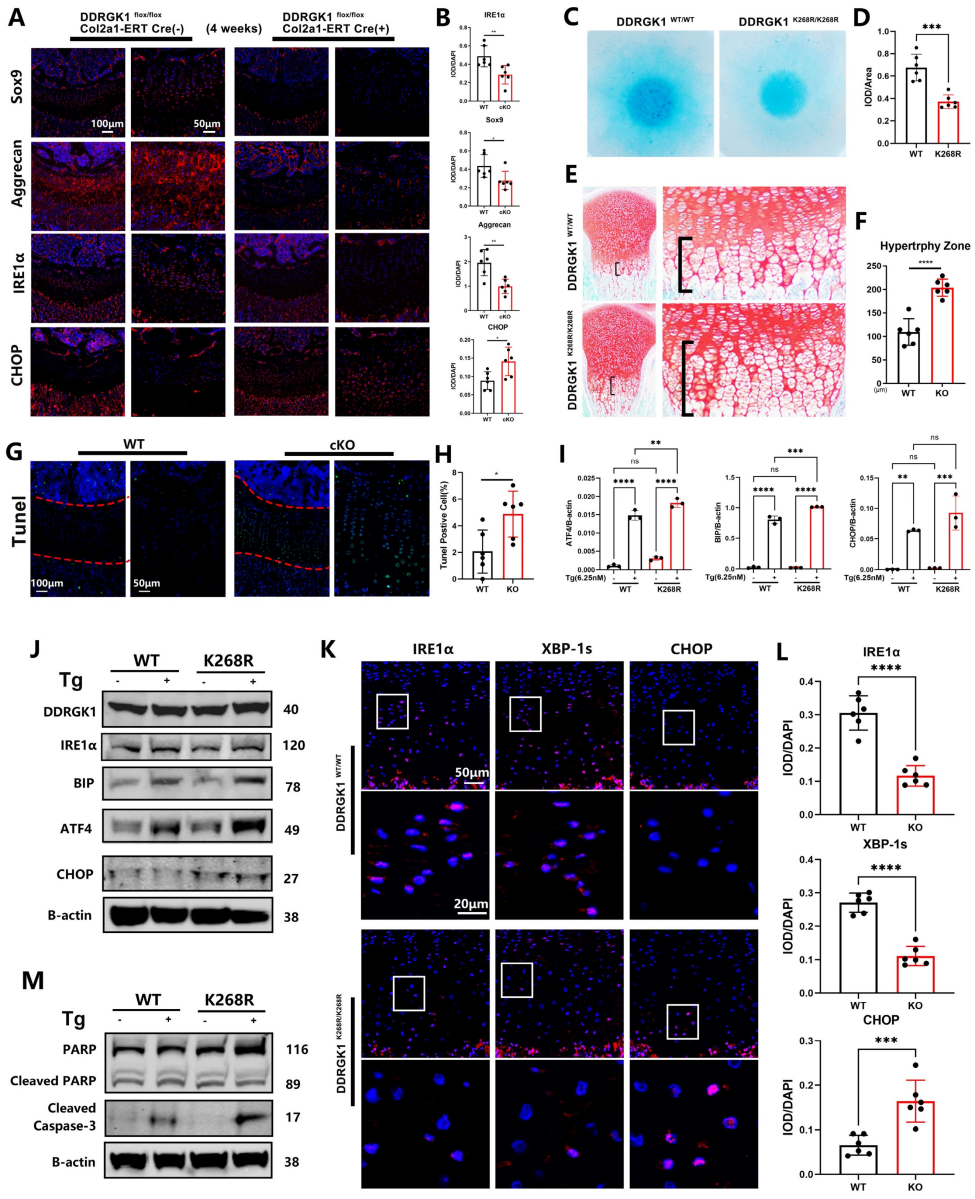
** Loss of DDRGK1 or the K268R mutation led to IRE1α degradation and ER stress aggravation in the growth plate cells of mice* in vivo.* (A)** Immunofluorescence analysis of SOX9, Aggrecan, IRE1α and CHOP expression in the lower limbs of the WT and cKO mice shown in Figure 1A; the growth plate is the focus. **(B)** Quantification of the integrated optical density (IOD)/DAPI stain intensity levels of SOX9, Aggrecan, IRE1α and CHOP in the images shown in panel (A). **(C)** Alcian blue staining of primary chondrocytes from WT and mutant mice 9 days after high-density culturing in chondrogenesis medium. **(D)** Quantification of the relative IOD/area ratio in the Alcian blue-stained cells shown in panel (C). **(E)** Safranin O-Fast Green staining of the lower limbs in WT and mutant mice; the growth plate is the focus. **(F)** Quantification of the length of the hypertrophy zone (HZ) shown in panel (E).** (G)** TUNEL immunofluorescence staining of the lower limbs of the WT and cKO mice shown in Figure 1A with focus on the growth plate. **(H)** Quantification of the percentage of TUNEL-positive cells in the growth plate shown in panel (G). **(I)** Reverse transcription-quantitative PCR analysis performed to determine the relative mRNA expression levels of ATF4, BIP and CHOP in primary WT and Mutant mouse chondrocytes treated with or without thapsigargin (Tg) for 24 h; β-actin was the internal reference. **(J)** Western blot analysis of DDRGK1, IRE1α, BIP, ATF4 and CHOP of primary WT and mutant mouse chondrocytes treated with or without Tg for 24 h; β-actin was the loading control. **(K)** Immunofluorescence analysis of IRE1α, XBP-1s and CHOP expression in the lower limbs of the WT and mutant mice shown in panel (E); the growth plate is the focus. **(L)** Quantification of the IOD/DAPI stain intensity levels of IRE1α, XBP-1s and CHOP in the chondrocytes shown in panel (K). **(M)** Western blot analysis of cleaved and full-length PARP and cleaved Caspase 3 in primary in WT and mutant mouse chondrocytes treated with or without Tg for 24 h; β-actin was the loading control. All data are presented as the mean ± SD from three or more experiments. ^*^P < 0.05, ^**^P < 0.01, ^***^P < 0.001 and ^****^P < 0.0001.

Performing immunofluorescence assay, we found that the levels of the chondrogenic markers Sox9, Aggrecan and Col2a1 were significantly decreased in DDRGK1-cKO mice compared with their levels in WT mice (**Figure 6A, B and Supplementary Figure 8a, b**), confirming a high knockout rate in the growth plate cells of the mice (**Supplementary Figure 8a, b**). The underlying mechanism might be the degradation of IRE1α and a subsequent increase in CHOP expression (**Figure 6A, B**), causing the increased hypertrophy and apoptosis observed in the HZ zone in a TUNEL assay (**Figure 6G, H**). Similar to the* in vivo* results, IRE1α expression was significantly reduced with CHOP expression significantly increased in the KO cell pellet compared with the NC cell pellet in immunofluorescence assays (**Supplementary Figure 8c, d**); these changes in IRE1α and CHOP expression disrupted chondrogenesis *in vitro*. As shown by TUNEL staining, the apoptotic rate (TUNEL-positive cells) of the KO cell pellet was significantly higher than that of the NC cell pellet (**Supplementary Figure 8e, f**).

Similar to the effect of the K267R mutation in the human DDRGK1 protein, the K268R mutation in the mouse DDRGK1 protein might damage the UFMylation system. Therefore, we established DDRGK1 K268R-mutant mice using the CRISPR/cas9 technique and performed further analyses. Alcian blue staining of primary chondrocytes showed impaired chondrogenesis of the DDRGK1 K268R-mutant cells (**Figure 6C, D**). We also observed an increase in the HZ in the K268R-mutant mice compared with the HZ in the WT mice (**Figure 6E, F**) *in vivo*. The underlying mechanism was the same as that of DDRGK1 knockout, in which the impaired UFMylation system led to decreased IRE1α expression, which was exacerbated with the addition of Tg, as shown by western blot and PCR assays (**Figure 6J and Supplementary Figure 9a**), followed by increased levels of ER stress pathway components, such as BIP, ATF4 and CHOP (**Figure 6I, J and Supplementary Figure 9b**). Increased ER stress caused cell apoptosis, and we observed that cleaved Caspase 3 was more highly expressed in K268R-mutant cells than in WT cells, although there was no change in the cleaved PARP levels (**Figure 6M and Supplementary Figure 9c**). Evaluating growth plate cells, we observed decreased expression of IRE1α and downstream XBP-1s in the DDRGK1 K268R-mutant mice compared with that in the WT mice (**Figure 6K, L**). Moreover, the expression of CHOP was increased in the K268R-mutant mice compared with that in the WT mice (**Figure 6K, L**).

## Discussion

Our study corroborated the suggestion that loss of DDRGK1 or mutation at a UFMylation site causes leads to a hypertrophic phenotype and apoptosis in growth plate cells, and the targeting efficiency was presented in the **Supplementary Figure 10a to 10d**, due to increased IRE1α instability, which activates the PERK/CHOP pathway and leads to apoptosis.

ER disruption can activate the UPR, which can in turn either mediate cell survival as ER homeostasis is gradually restored or can trigger apoptosis when the UPR is chronically stimulated or when protein accumulation is excessive 30-32. There are three main sensory receptors on the surface of the ER, namely, IRE1α, PERK and activating transcription factor 6 33-35. During early UPR stages, IRE1α splices the mRNA of the transcription factor X-box-binding protein 1 to induce the transcription of ER quality control components, such as XBP1, that restore ER homeostasis 29, 36 and promote cell survival. In our study, we found that loss or mutation of DDRGK1 caused decreased protein expression of IRE1α, which disrupted the self-promoting survival cycle. When IRE1α fails to maintain homeostasis, c-Jun N-terminal kinase signaling induces apoptosis 37. PERK directly phosphorylates the subunit of eukaryotic initiation factor 2, which selectively translates and activates transcription factor 4 to upregulate the expression of a multitude of UPR target genes, including those involved in ER stress-mediated apoptosis, such as CHOP 38.

DDRGK1 has been identified as critical IRE1α modification via UFMylation 39. Moreover, DDRGK1 is not only a substrate in UFMylation but is also a cofactor in UFMylation 9, 40, 41. DDRGK1 has been reported to facilitate protein UFMylation to maintain protein stability in a number of diseases, including uterine endometrioid carcinoma, neurodegenerative diseases, and developmental defects 10, 42, 43. Therefore, in our study, a connection between DDRGK1 and the ER was revealed, with DDRGK1 facilitating the UFMylation of IRE1α to maintain IRE1α expression and activity *in vivo* and* in vitro*. Previous studies have shown that DDRGK1 regulates the stability of IRE1α in several types of cancer cells, such as MCF-7 and HepG2 cells 20. Our observations suggested that deletion of DDRGK1 leads to reduced UFMylation of IRE1α in *Ddrgk1*^fl/fl^, *Col2a1*-ERT Cre mice *in vivo* and in ATDC5 chondrocytes *in vitro*. DDRGK1 knockout increased the ubiquitylation-mediated degradation of IRE1α, subsequently activating the UPR and inducing apoptosis.

Apoptosis is a form of programmed cell death that plays key roles under pathological conditions 44-46. Although it can be a negative feedback mechanism during tissue development and regeneration 47, apoptosis can be dysregulated, which results in various types of complex abnormalities 48. In our study, we observed an increase in the HZ in DDRGK1-cKO mice in different time periods ranging from E17.5 to the 4th neonatal week, although there is no dwarf phenotype; however, we observed a decreased growth plate length in cKO mice at 9 weeks, these results combined mean that the dwarf phenotype occur and exacerbated over time. Combined with these* in vivo* and *in vitro* data and considering that previous studies have proven that normal programmed apoptosis of HZ cells in the growth plate is a critical process in mouse development 49-51, instead of the critical and classical pathways to modulate SOX9, our study provided a new opinion to elucidate the mechanism of SEMD caused via DDRGK1 mutation. We believed that the apoptotic pathway is contributed to, at least partially, the defect of the chondrogenesis in patients, as well as SOX9 modulation. This supposition was further proved by DDRGK1 K268R-mutant mice *in vivo* and chondrocytes *in vitro*.

In conclusion, in the present study, we mainly focused on the mechanism involved in SEMD, and the relationships among apoptosis, cartilage differentiation and ER stress were congruently deciphered. With *Ddrgk1*-cKO mice and ATDC5 chondrocytes, a DDRGK1/UFMylation system was discovered and characterized, promoting the degradation of IRE1α and the ensuing CHOP/apoptosis activation but not the coactivation of pyroptosis, apoptosis and necrosis. This process may underlie at least in part the growth retardation during SEMD pathogenesis, which includes abnormal spinal development 52. The data reported in this study may lead to novel gene targets for future SEMD treatment. For patients suffering from SEMD, the DDRGK1 gene should be the focus as a potential target, and from the perspective of gene treatment, DDRGK1-targeted gene therapy may be used in a novel approach in the future.

## Materials and methods

### Reagents

Polymerase chain reaction (PCR) primers (Sangon Biotech Co., Ltd., Shanghai, China), Cell Counting Kit-8 (CCK-8; Dojindo Molecular Technologies, Inc., Kumamoto, Japan), TRIzol^®^ reagent (Invitrogen; Thermo Fisher Scientific, Inc., Carlsbad, CA, U.S.A), PrimeScript™ RT Master Mix Kit (036a, Takara Biotechnology Co., Ltd., Dalian, China), TB Green® Premix Ex Taq™ Kit (420a, Takara Biotechnology Co., Ltd., Dalian, China), Phosphorylase Protease Inhibitor Mixture (Thermo Fisher Scientific, Inc., Waltham, Ma, U.S.A), DAPI (Sigma Aldrich, Merck KGaA, St Louis, MO, U.S.A), PVDF membranes (EMD Millipore, CA, U.S.A)Primary antibodies against myc-tag (cat. no. 71d10; rabbit mAb), B-cell lymphoma 2 (Bcl-2)-associated X (Bax; cat. no. d3r2m; rabbit mAb), IRE1α (cat. no. 14c10; rabbit mAb), XBP-1s (cat. no. E9V3E; rabbit mAb), protein disulfide isomerase (cat. no. c81h6; rabbit mAb), binding immunoglobulin protein (BiP; cat. no. c50b12; rabbit mAb), C/EBP homologous protein (CHOP; cat. no. l63f74; mouse mAb), Protein kinase RNA-like endoplasmic reticulum kinase (PERK; cat. no. c33e10; rabbit mAb), Caspase 3 (cat. no. 9662; rabbit mAb), Caspase 9 (cat. no. 9508; rabbit mAb), Cleaved Caspase 9 (cat. no. 9507; rabbit mAb), poly (ADP-ribose) polymerase (PARP; cat. no. 46d11; rabbit mAb) and β-actin (cat. no. d6a8; rabbit mAb) were purchased from Cell signaling Technology, Inc., Denver, MA, USA. Anti-IRE1α (cat. no. ab37073; rabbit mAb), Anti-gasdermin D (GSDMD; cat. no. ab219800; rabbit mAb), Anti-receptor-interacting protein (RIP; cat. no. ab202985; rabbit mAb), Anti-SOX9 (cat. no. ab185966; rabbit mAb) and Anti-UFM1 (cat. no. ab109305; rabbit mAb) were purchased from Abcam, Cambridge, UK. Anti-DDRGK1 (cat. no. 21445-1-AP; rabbit mAb) primary antibodies were purchased from ProteinTech Group, Inc., Chicago, IL, USA. Anti-Bcl-2 (cat. no. AF6139; rabbit pAb) and Anti-aggrecan (cat. no. DF7561; rabbit pAb) were purchased from Affinity Biosciences, Changzhou, China. Anti-hemagglutinin (HA)-tag (cat. no. abs137982; rabbit pAb) and anti-FLAG tag (cat. no. abs120265; rabbit pAb) primary antibodies were purchased from Absin Bioscience, Inc., Shanghai, China.

### Animal Experiments

*Ddrgk1*^fl/fl^ mice, *Col2a1*-ERT Cre mice and DDRGK1^K268R/K268R^ mice were generated or purchased from the Gempharmatech Co., Ltd, Jiangsu, China. Then *Ddrgk1*^fl/fl^ mice was crossed with *Col2a1*-ERT Cre mice to generate *Ddrgk1*^fl/fl^, *Col2a1*-ERT Cre (+) mice and their littermate control mice. For the *Ddrgk1*^fl/fl^, *Col2a1*-ERT Cre (+) mice sacrificed at P28, the time point of Tamoxifen induction (one time, 10μl, 12.5mg/ml in corn oil, intragastric injection) is at the P3; For the infant mice of E17.5, P3 and P10, the time point of induction (one time, 70mg/kg in corn oil, intraperitoneal injection) is at the E11.5 for E17.5, and at the E13.5 for P3 and P10 through Tamoxifen administration to maternal mice (*Ddrgk1*^fl/fl^, *Col2a1*-ERT Cre (-));For the *Ddrgk1*^fl/fl^, *Col2a1*-ERT Cre (+) mice sacrificed at P63, the time point of Tamoxifen induction (five times, 70mg/kg in corn oil, intraperitoneal injection) is at the P28-32. For the growth curves analysis, the time point of induction is at the E13.5 through Tamoxifen administration to maternal mice (*Ddrgk1*^fl/fl^, *Col2a1*-ERT Cre (-)), after birth, the mass and length of infant mice were measured every two days until P31.

We make *Ddrgk1* point mutation (mutant site is K268R) mice via CRISPR/Cas9 system. Firstly, one sgRNA-targeting Exon17 of *Ddrgk1* gene were respectively constructed and transcribed* in vitro*. And the donor vector with the *Ddrgk1* fragment was designed and constructed *in vitro*. Then Cas9 mRNA, sgRNA and donor will be co-injected into zygotes. Thereafter, the zygotes were transferred into the oviduct of pseudo-pregnant ICR females at 0.5 dpc. And F0 mice was birthed after 19~21 days of transplantation, all the offspring of ICR females (F0 mice) were identified by PCR and sequencing of tail DNA. And positive F0 mice which had a copy of the point mutation of *Ddrgk1* gene were genetyped by the methods. Finally, crossing F0 mice with C57BL/6J mouse to build up heterozygous mice.

C57BL/6J background were maintained during housing and mice were raised under specific-pathogen-free (SPF) conditions in the Department of Laboratory Animal Science at Shanghai Ninth People's Hospital. Mice were housed under pathogen-free conditions at 26-28°C and 50-65% humidity with 12-hour day/night cycle. Animals were fed standard rodent chow and had access to fresh water *ad libitum*. All animal experimentation were approved by the Institutional Animal Care and Ethics Committee of Ninth People's Hospital, Shanghai Jiaotong University School of Medicine (Shanghai, China) and performed in accordance with the principles and procedures of the National Institutes of Health (NIH) Guide for the Care and Use of Laboratory Animals and the Guidelines for Animal Treatment of Shanghai Jiaotong University.

### Isolation and culture of primary chondrocytes in mice

Three 5-day old WT and K268R, WT and cKO mice range from 2-4g (Shanghai Lab, Animal Research Center Co., Ltd) were sacrificed via decapitation, immersed in the 75% ethanol for 10 min. The lower limbs were dissected, the skins were removed, and then the whole knee joints (like two glassy drops) were extracted with the synovial tissue and muscle tissue stripped, then these drops were cut into pieces ranged from 0.5 mm to 1 mm and soaked in 1% collagenase II solution for 2 h, followed by centrifugation in 0.3 g, 37 ℃ for 5mins and suspension. The primary chondrocytes were cultured in DMEM/F12 (Gibco; Thermo Fisher Scientific, Inc.) supplemented with 5% FBS, 1% penicillin-streptomycin (Gibco; Thermo Fisher Scientific, Inc.) and 1% ITS solution.

### Culture of ATDC5 cells and 293T cells

ATDC5 chondrocytes, 293T cells are immortalized cell lines purchased from Shanghai Fuheng Biological Company (cat. no. FH0378, FH0244). ATDC5 were cultured in Dulbecco's modified Eagle medium F12 (DMEM/F12) with 5% fetal bovine serum (FBS) and 1% penicillin and streptomycin (Gibco, Thermo Fisher Scientific, Inc., Waltham, MA, USA) at 37℃ with 5% CO^2^. 293T were cultured in Dulbecco's modified Eagle's medium (DMEM) supplemented with 10% FBS and 1% penicillin-streptomycin (Gibco, Thermo Fisher Scientific, Waltham, MA, USA) at 37˚C with 5% CO_2_.

### DDRGK1 knockout in ATDC5 cells

DDRGK1 knockout were produced using the NC (GCACTACCAGAGCTAACTCA; pLenti-U6-spgRNA v2.0-CMV-EGFP) and KO (CCCCGGCGTCGGAGGGACTT; pLenti-U6-spgRNA v2.0 (Ddrgk1)-CMV-EGFP) and Cas9 virus (pLenti-CMV-Puro-P2A-3Flag-espCas9_1.1), were purchased from Shanghai Heyuan Biological Company, Shanghai, China. For transfection, ATDC5 chondrocytes were seeded into a six-well plate at a density of 1x 10^5^ cells per well. On day 2, Cas9 virus were added with a MOI of 20. On day 3, the media was changed, and the cells were screened with puromycin (cat. no. BS111; Biosharp, Hefei, China) at a concentration of 5 μg/ml. On day 5, the Cas9 cells were then transfected with the NC or KO viruses with a MOI of 20 for the second transfection. Among them, on day 7 the Cas9/NC and Cas9/KO cell lines were then subjected to monoclonal culture. Firstly, 100 µl DMEM/F12 medium with 5% FBS and 1% penicillin and streptomycin were added to each well in a 96-well plate before 1,000 cells in 100 µl DMEM/F12 medium were added to A1 and mixed thoroughly. Subsequently, 100 µl DMEM/F12 medium with cells was transferred from A1 to B1, then repeatedly into H1. We then used an eight-channel pipette to transfer 100 µl medium with cells from the first line to the second line and finally into the last line in a 1:1 ratio. The cells were cultured in DMEM/F12 medium with 10% FBS and 1% penicillin and streptomycin at 37℃ with 5% CO^2^.

### High-density culture and pellet culture

To evaluate chondrocyte differentiation, 1.5 x 10^5^ ATDC5 cells or primary chondrocytes were resuspended in 10 μl medium and seeded into a 24-well plate. Cells were allowed to adhere at 37˚C for 1 h, before 0.5 ml DMEM/F12 medium containing 10 ng/ml insulin-transferrin-selenium (ITS) and 2% FBS was added. After 24 h at 37˚C, the cells were stimulated with or without thapsigargin (Tg, 6.25 nM; Apexbio; cat. no. B6614; Houston, TX, USA) and the medium was changed every 2 days. After 9 days at 37˚C, the micromasses were stained with alcian blue for 24 h at room temperature (RT).

For the pellet culture, 1.5 x 10^7^ ATDC5 cells or primary chondrocytes were centrifuged as a pellet in the bottom of a 15-ml centrifuge tube, which was filled with the Mesenchymal Stem Cell Chondrogenic Differentiation Medium (Cyagen Biosciences, Inc., Santa Clara, CA, USA). The medium was refreshed every 3 days. After 21 days of culture at 37˚C, the pellets were collected via tweezer and fixed in 4% paraformaldehyde (PFA) for 5 h at RT and then embedded in optimal cutting temperature compound (Sakura Finetek USA, Inc.). The pellets were then stored at -80˚C overnight and cut to a 20-μm thickness using a freezing microtome (Leica Microsystems GmbH).

### RNA extraction and reverse transcription-quantitative PCR (qPCR) analysis

According to the manufacturer's protocols, TRIzol Reagent was used to isolate total RNA from tissues and cells. First strand complementary DNA (cDNA) was reverse transcribed from the extracted RNA using a PrimeScript™ RT Master Mix Kit (036a, Takara Biotechnology Co., Ltd., Dalian, China). TB Green Premix Ex Taq Kit (Takara bio, Inc., Otsu, Japan) was used to perform qPCR in the Applied Biosystems QuantumStudio 6 Flex Real-Time PCR system (Thermo Fisher Scientific, Inc., Waltham, MA, USA) per the following conditions: Denaturation at 95˚C for 30 sec; 40 cycles of 95 ˚C for 3 sec and 60 ˚C for 34 sec; and then 95 ˚C for 15 sec, 60 ˚C for 60 sec and finally, 95 ˚C for 15 sec. Specific primer pairs were designed using NCBI blast and the sequences are provided in Table S1. GAPDH gene expression was used as the internal controls. Target gene expression levels were determined using the 2^-ΔΔCq^ method 53.

### RNA seq analysis

Total RNA of NC or KO cells were extracted using TRIzol reagent (Thermo Fisher Scientific, Waltham, MA, USA) as per manufacturer's protocol and analyzed via RNA (transcriptome) sequencing as Wuhan Huada Gene Technology Co., Ltd. (China): Volcano Plot (|log2FC| >= 1, FDR <= 0.001), Kyoto Encyclopedia of Genes and Genomes (KEGG) pathways, Gene Set Enrichment Analysis* (*GSEA) and Heat Map analysis were used by mRNA relative expression as Transcripts PerKilobase Million (TPM) to further review pathways involved on the Mybgi platform (WuhanHuada Gene Technology, https://mybgi.bgi.com/tech/login).

### Cell viability test

CCK-8 was used to evaluate cell viability of DDRGK1 NC and KO ATDC5 cells. The cells were seeded into a 96-well plate at a density of 3,000 cells per well the day before treatment with thapsigargin (Tg, 6.25 nM; Apexbio; cat. no. B6614; Houston, TX, USA) for 24, 48, 72 and 96 h at 37˚C. ATDC5 chondrocytes were cultured in DMEM/F12 supplemented with 5% FBS and 1% penicillin and streptomycin at 37℃ with 5% CO^2^. The cell culture medium was changed every 2 days. At the end of the experiment, fresh 100 μl medium containing 10 μl CCK-8 reagent was added into each well prior to incubation at 37˚C for 1 h. Medium containing the CCK-8 reagent added to wells without cells was designated as the blank group whereas untreated cells were designated as the control group. Absorbance at 450 nm (as measured by optical density; OD) in each well was measured using the Infinite M200 Pro microplate reader (Tecan Group, Ltd., Mannedorf, Switzerland).

### Co-immunoprecipitation (Co-IP) assay

293T cells or NC and KO ATDC5 chondrocytes were transfected with Flag-PLVC, Flag (HA)-DDRGK1, Flag-DDRGK1 K267R, HA (Flag)-IRE1α NC, HA (Flag)-IRE1α ΔC, HA (Flag)-IRE1α ΔN, HA (Flag)-IRE1α luminal domain (LD), HA (Flag)-IRE1α cytosolic domain (ICD), Flag-IRE1α Del468, Flag-IRE1α Del469 and HA-UFM1 plasmids (synthesized, purchased from Shanghai Ai Bosi Biological Technology Co., Ltd.) using lipofectamine 3000 (cat. no. L3000015; Thermo Fisher Scientific, Inc., Waltham, MA, USA) before they were washed three times with phosphate-buffered saline (PBS). Subsequently, 1.2 ml RIPA lysis buffer with 12 μl protease inhibitor, protein phosphatase inhibitor A + B and PMSF (Roche Diagnostics GmbH, Mannheim, Germany) was added to the cells and incubated for 20 min at 4˚C. After centrifugation at 13000 x g for 15 min at 4˚C, to 200 μl of the protein supernatant 50 μl protein loading buffer (5X) was added and this sample was boiled at 99˚C for 10 min whereas the remaining 1,000 μl of the protein supernatant was incubated with 30 μl Flag-tagged (or HA-tagged) magnetic beads (Sigma-Aldrich, Merck KGaA, St Louis, MO, U.S.A.) at 4˚C overnight. The next day, the magnetic beads were isolated from protein supernatant by Magnet Frame (Selleck Chemicals, China), and then washed three times with Tris-buffered saline (TBS) at 4˚C for 10 min with protease inhibitor, protein phosphatase inhibitor A + B and PMSF before being boiled at 99˚C for 5 min with 50 μl 1X RIPA lysis bufferto extract the proteins.

### UFM1 modification assay

293T cells were transfected with Flag-PLVC, Flag-IRE1α, HA-UFM1, Myc-DDRGK1, Myc-UFM1 specific ligase 1 (UFL1) and Myc-UFM1-conjugating enzyme 1 (UFC1) or were transfected with Flag-PLVC, Flag-IRE1α, HA-UFM1, si-DDRGK1, Myc-UFL1 and Myc-UFC1 plasmids (synthesized, purchased from Shanghai Ai Bosi Biological Technology Co., Ltd.) using lipofectamine 3000 (cat. no. L3000015; Thermo Fisher Scientific, Inc., Waltham, MA, USA). After culturing for 48 h, cells were washed three times with PBS before the protein samples were extracted using 1.2 ml NETT. In total, 200 μl lysate was used as input and the remaining 1,000 μl was incubated with 30 μl Flag-tagged magnetic beads at 4˚C overnight. The next day, the beads were boiled with 50 μl 1X loading RIPA lysis buffer at 99˚C for 10 mins and finally subjected to 10 or 12.5% SDS-PAGE electrophoresis buffer followed by immunoblot analysis.

### Ubiquitylation modification assay

293T cells were transfected with Flag-PLVC, HA-IRE1α and Flag-Ubiquitin, Flag-PLVC and Flag-Ubiquitin or with Flag-PLVC, Flag-IRE1α, HA-UFM1, Myc-DDRGK1, Myc-UFL1, Myc-UFC1 and Flag-Ubiquitin plasmids (synthesized, purchased from Shanghai Ai Bosi Biological Technology Co., Ltd.) using lipofectamine 3000 (cat. no. L3000015; Thermo Fisher Scientific, Inc., Waltham, MA, USA). After culturing for 40 h, cells were stimulated with MG132 (10μM; cat. no. S2619; Selleck Chemicals, China) for 8 h at 37˚C and then washed three times with PBS. After using 1.2 ml NETT to extract protein, 200 μl lysate was used as input whilst the remaining 1,000 μl samples were incubated with 30 μl HA-tagged magnetic beads at 4˚C overnight. The samples were then boiled at 99˚C for 10 min and finally subjected to 10 or 12.5% SDS-PAGE electrophoresis buffer followed by immunoblot analysis.

### Hoechst stain for apoptosis

ATDC5 cells or primary chondrocytes were seeded into a six-well plate at a density of 3 x 10^4^ cells per well. The next day, cells were fixed with 4% PFA for 5 min at RT and washed with PBS three times. Hoechst stain solution (5 μg/ml; Beyotime Institute of Biotechnology) were added for 5 min at RT and washed with PBS for three times, before the cells were imaged using fluorescence microscopy (Leica Microsystems GmbH) at x10 and x20 magnification.

### Flow cytometry

According to the manufacturer's protocol, flow cytometry was used to evaluate the effect of 1x 10^6^ ATDC5 cells or primary chondrocytes on the apoptosis rate after staining with Annexin V, 633 Apoptosis Detection Kit (cat. no. AD11; Dojindo Molecular Technologies, Inc., Kumamoto, Japan). FACSCalibur Flow Cytometer (BD Biosciences) was used to count ≥ 20,000 events. The percentage of cells in the upper right quadrant (Q2; annexin V and PI positive) and the lower right quadrant (Q3: annexin V positive and PI negative) was used to quantify the apoptosis rate.

### Western blotting

Total protein was extracted from the ATDC5 cells or primary chondrocytes using RIPA lysis buffer supplemented with phosphatase and protease inhibitors (Roche, Basel, Switzerland). The protein was quantified by BCA assay (Thermo Fisher Scientific, Inc.) and then equal amounts of the extracted protein were separated (20-30 μg) in a 4-20% SurePAGE™ gel and transferred onto 0.22 μm PVDF membranes (MilliporeSigma). The membranes were blocked with 5% bovine serum albumin (BSA)-TBS-Tween 20 (TBST) at room temperature for 1 h and then incubated with primary antibodies (diluted 1:1,000 in 5% BSA) against myc-tag, Bax, IRE1α, PDI, BiP, CHOP, PERK, Caspase 3, Caspase 9, Cleaved Caspase 9, PARP, β-actin, GSDMD, RIP, SOX9, UFM1, DDRGK1, Bcl-2, Aggrecan, HA-tag, FLAG-tag at 4˚C overnight. The next day, all membranes were washed with TBST and incubated with the anti-rabbit IgG (H + L) (dylight)™ secondary antibody (cat. no. 5151; DyLight™ 800 4X PEG Conjugate; Cell Signaling Technology, Inc.; 1:5,000) at room temperature for 1 h in the dark. The membranes were extensively washed in TBST before the protein bands were detected using a Li-Cor Odyssey Fluorescence Imaging System (Li-COR Biosciences, Lincoln, NE, USA). Intensity of the protein immunoreactive bands was measured using the Image Pro Plus 6.0 software (Media Cybernetics, Inc.) with intensity of the β-actin band used as internal reference.

### Immunofluorescence staining

The pellet culture sectioned into 20-μm thick frozen sections for histological evaluation, and the tibia bones were embedded into paraffin blocks then subjected to histological sectioning (5-μm thickness). These sections were washed with PBS three times at RT to remove the OCT solution and stained with Safranin O-Fast green (cat. no. G1053; Servicebio, Wuhan, China) and hematoxylin and eosin dyes (cat. no. G1001; Servicebio, Wuhan, China) at RT for 2-5 min, in accordance with the manufacturer's protocols.

For immunofluorescence staining, the frozen sections were defrosted at room temperature for 30 min, alone with paraffin sections de-paraffinized and antigen-retrieved, washed with PBS three times and incubated at 37˚C in an antigen retrieval buffer (Roche Diagnostics, Basel, Switzerland) for 30 min. An auto-fluorescence quenching agent was added to the sections for 5 min at RT and blocked with blocking buffer at room temperature for 30 min. The sections were then incubated with primary antibody (1:100 dilution) IRE1α (cat. no. ab37073; rabbit mAb), XBP-1s (cat. no. E9V3E; rabbit mAb), SOX9 (cat. no. ab185966; rabbit mAb), Col2a1 (cat. no. ab34712; rabbit mAb), CHOP (cat. no. l63f74; mouse mAb), DDRGK1 (cat. no. 21445-1-AP; rabbit mAb) and Anti-aggrecan (cat. no. DF7561; rabbit pAb) in a wet box at 4˚C overnight. The next day, the slices were washed with PBS and incubated with the Alexa-Fluor^®^ 594-conjugated secondary antibody (cat. no. 8889; anti rabbit; 1:500; Cell Signaling Technology, Inc., Danvers, MA, USA) in the dark at room temperature for 50 min. The sections were washed with PBS and incubated with a DAPI solution (Sigma Aldrich, Merck KGaA, St. Louis, MO, USA) in the dark for 10 min at RT to stain the nuclei. After a final wash with PBS, the samples were air-dried and sealed with anti-fluorescence quenching tablets. A fluorescence image was then taken using the Leica DM4000 B fluorescence microscope (Leica Microsystems GmbH) at a x10 magnification, and the integrated optical density (IOD)/DAPI was measured by Image Pro Plus 6.0 software (Media Cybernetics, Inc.).

### TUNEL assay

The pellet frozen sectons and tibia paraffin section were washed with PBS three times at RT and stained with Fluorescein (FITC) Tunel Cell Apoptosis Detection Kit (cat. no. G1501; Servicebio, Wuhan, China) according to the manufacturer's protocols. A fluorescence image was then taken using the Leica DM4000 B fluorescence microscope (Leica Microsystems GmbH) at a x10 magnification, and the TUNEL-positive cells was measured with the following formula: TUNEL-positive cells/total number of cells × 100% by Image Pro Plus 6.0 software (Media Cybernetics, Inc.).

### Radiographic analysis

Digital X-ray imaging of the whole mice was conducted in the anteroposterior axis with a 21 lp/mm detector that provides up to x5 geometric magnification, the photos were taken under 28.3kV and 8.2 ms (Faxitron VersaVision; Faxitron Bioptics LLC).

### Statistical analysis

Three independent experiments were performed on all data. The data were expressed as the mean ± standard deviation. SPSS 19.0 software (IBM Corp, Armonk, NY, USA) was used to perform two-tailed unpaired Student's t-test or one-way ANOVA with Tukey's post hoc test on the data. Unless otherwise specified, P < 0.05 was considered to indicate a statistically significant difference.

## Supplementary Material

Supplementary figures and table.

## Abbreviations

SEMD: Spondyloepiphyseal dysplasia
UPR: unfolded protein response
ER: endoplasmic reticulum membrane
UFBP1: UFM1 binding protein 1 with PCI domain
ERα: estrogen receptor α
ASC1: activating signal cointegrator 1
SOFG: safranin o-fast green
IOD: integral optical density
BAX: BCL2 associated X, apoptosis regulator
BCL2: B cell leukemia/lymphoma 2 apoptosis regulator
PARP: Poly-(ADP-ribose) polymerase
GSDMD: gasdermin D
RIP: regulation of phenobarbitol-inducible P450
ECM: extracellular matrix
Col2a1: collagen type II alpha 1 chain
CHOP: C/EBP homologous protein
CHX: Cycloheximide
Chlq: chloroquine
WCL: whole cell lysate
Tg: Thapsigargin
BIP: ER luminal binding protein precursor
PERK: PKR-like endoplasmic reticulum kinase
SOX9: SRY (sex determining region Y)-box 9
RUNX2: runt related transcription factor 2
MYOD: myogenic differentiation
TAL1: T cell acute lymphocytic leukemia 1
CMD: myelodysplasia
ATF6: activating transcription factor 6
XBP1: X-box binding protein 1
JNK: mitogen-activated protein kinase 9
eIF2a: eukaryotic initiation factor 2
ATF4: activates transcription factor 4
UBA5: ubiquitin like modifier activating enzyme 5
UFL1: UFM1 specific ligase 1
UFC1: ubiquitin-fold modifier conjugating enzyme 1
CDK5RAP3: CDK5 regulatory subunit associated protein 3
UFM1: ubiquitin fold modifier 1
