# Supplementary material for: Loss of DDRGK1 impairs IRE1α UFMylation in spondyloepiphyseal dysplasia
Source: Int J Biol Sci. 2023 Sep 4;19(15):4709–25. doi: 10.7150/ijbs.82765 (PMC10539710; doi:10.7150/ijbs.82765)
Supplement: Supplementary file 1 — Supplementary figures and table. [file ijbsv19p4709s1.pdf]

Supplementary Information

Loss of DDRGK1 impairs IRE1α UFMylation in spondyloepiphyseal dysplasia

Xiao Yang[1†], Tangjun Zhou[1†], Xin Wang[1†], Ying Xia[2], Xiankun Cao[1], Xiaofei Cheng[1], Yu Cao[1,2], Peixiang Ma[1], Hui Ma[1*], An Qin[1*], and Jie Zhao[1*]

[1]Shanghai Key Laboratory of Orthopedic Implants, Department of Orthopedics, Ninth People's Hospital, Shanghai Jiao Tong University School of Medicine, Shanghai, China
[2]Institute of Precision Medicine, The Ninth People's Hospital, Shanghai Jiao Tong University School of Medicine, Shanghai, China

[†]These authors have contributed equally to this work and share first authorship.

Email addresses:
Xiao Yang
walkingweapon@outlook.com
Tangjun Zhou
zhoutangjun@outlook.com
Xin Wang
walsheim@163.com
Ying Xia
xiaying@shsmu.edu.cn
Xiankun Cao
lemonwilson@sjtu.edu.cn
Xiaofei Cheng
drcxfhy@163.com
Yu Cao
Yu.Cao@shsmu.edu.cn
Peixiang Ma
mapx@shsmu.edu.cn

***Correspondence:**
Hui Ma
orthopain@163.com
An Qin
dr_qinan@163.com
Jie Zhao
profzhaojie@126.com

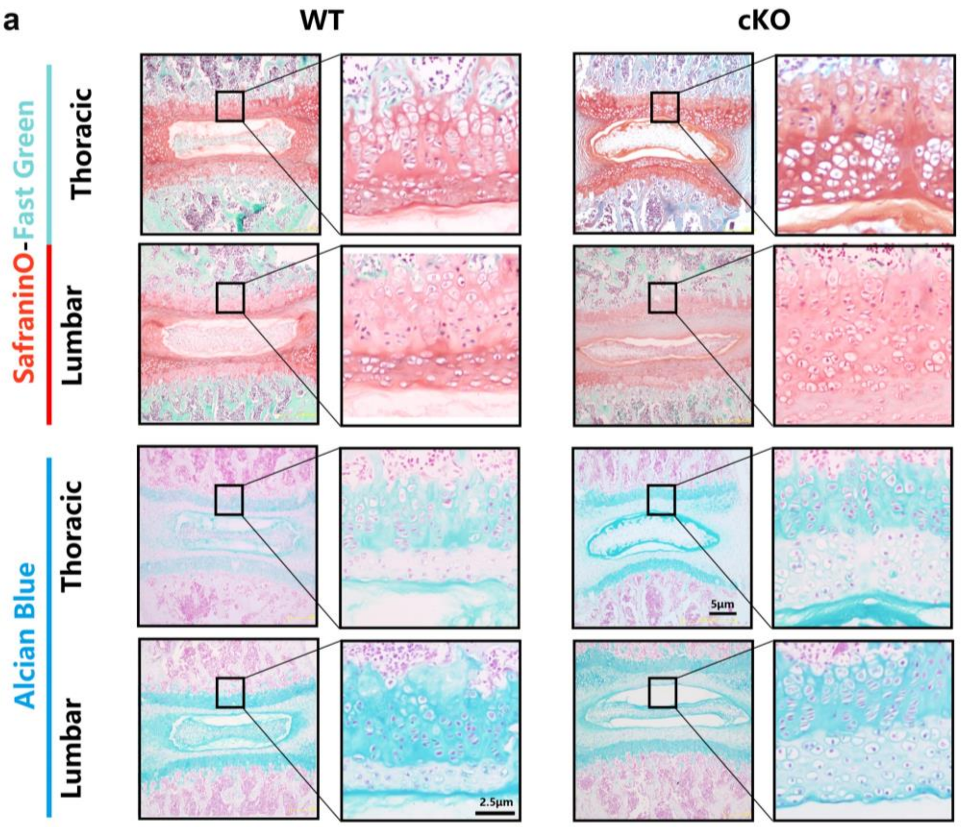

Supplementary Figure 1 | (a) Safranin O-Fast Green staining and Alcian blue staining of lumbar and thoracic spine in WT and cKO mice shown in Figure 1A and focus on the area of growth plate.

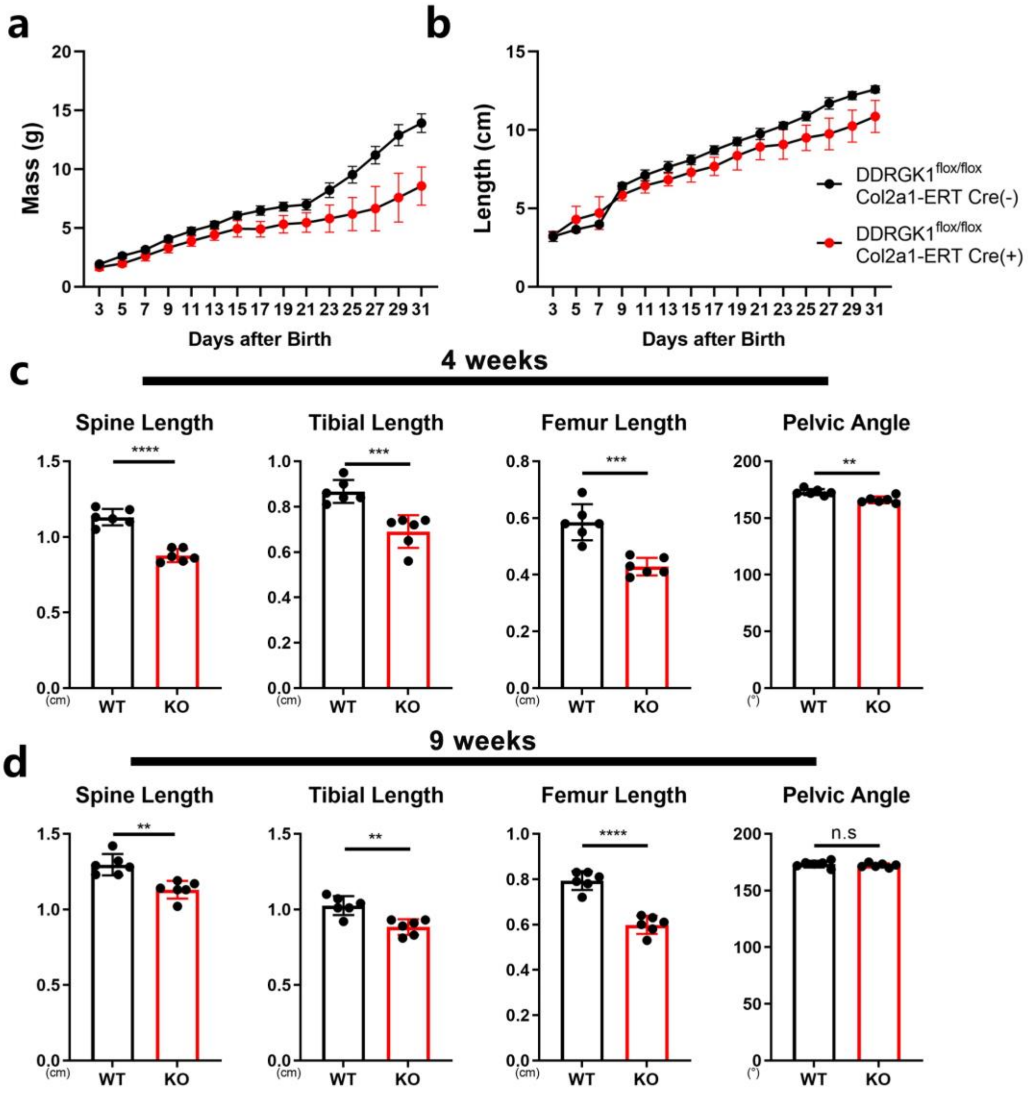

Supplementary Figure 2 | (a, b) Growth curves of mass and length in WT and cKO mice until 31 days after birth. (c) Quantification of the tibial length, spine length, femur length and pelvic angle of the female WT and cKO mice in 4 weeks. (d) Quantification of the tibial length, spine length, femur length and pelvic angle of the female WT and cKO mice in 9 weeks.

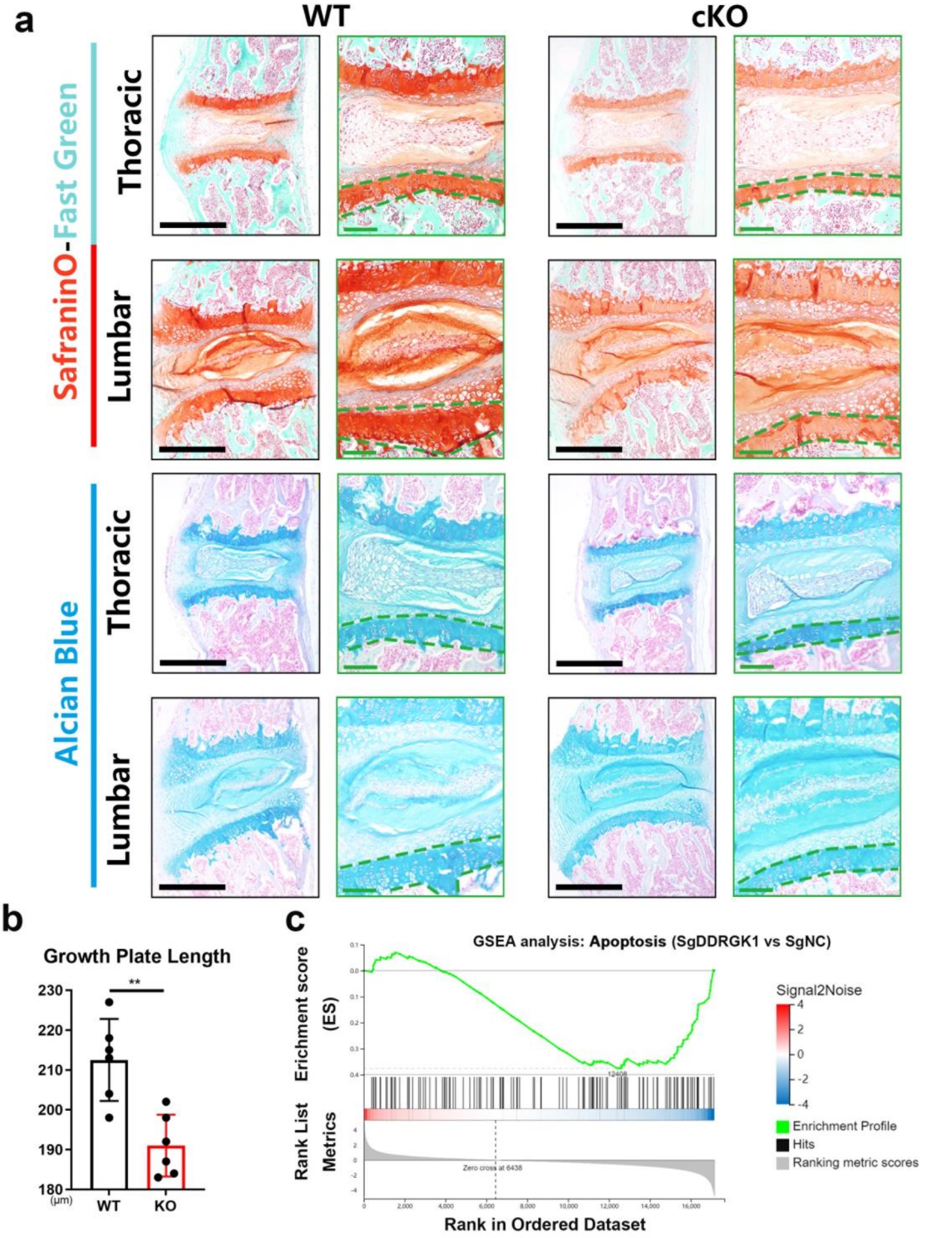

Supplementary Figure 3 | (a) Safranin O-Fast Green staining and Alcian blue staining of lumbar and thoracic spine in WT and cKO mice shown in Figure 2A and focus on the area of growth plate. (b) Quantification of the length of growth plate shown in Figure 2B. (c) GSEA analyses of Apoptosis pathways in SgNC and SgDDRGK1 ATDC5 chondrocytes shown in Figure 3A.

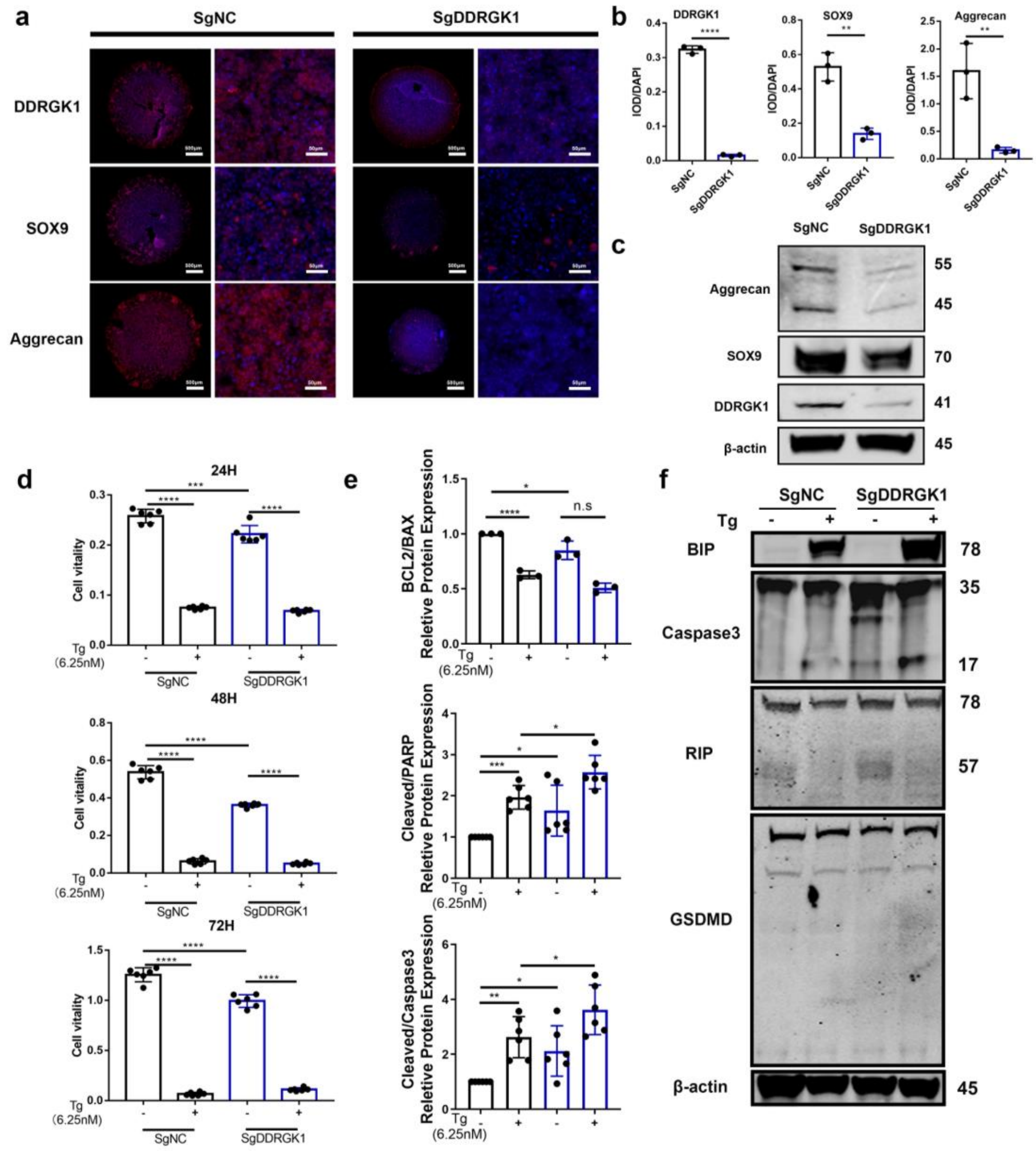

Supplementary Figure 4 | (a) Immunofluorescence analysis of DDRGK1, SOX9 and aggrecan expression in the pellet culture of SgNC and SgDDRGK1 ATDC5 chondrocytes 21 days after culture in chondrogenesis medium. (b) Quantification of the IOD/DAPI levels of DDRGK1, SOX9 and Aggrecan expression shown in panel (a). (c) Western blot analysis of aggrecan, SOX9 and DDRGK1 expression using β-actin as the loading control in SgNC and SgDDRGK1 ATDC5 chondrocytes. (d) Cell viability of SgNC and SgDDRGK1 ATDC5 chondrocytes with or without Tg (6.25 nM) treatment for 24, 48 and 72 h. (e) Quantification of the gray values as the ratio of Bax/Bcl2, cleaved/total Caspase 3 and cleaved/total PARP in SgNC and SgDDRGK1 ATDC5 chondrocytes shown in Figure 3G. (f) Western blot analysis of BiP, cleaved/total Caspase 3, RIP and GSDMD using β-actin as the loading control in SgNC and SgDDRGK1 ATDC5 chondrocytes treated with Tg for 24 h.

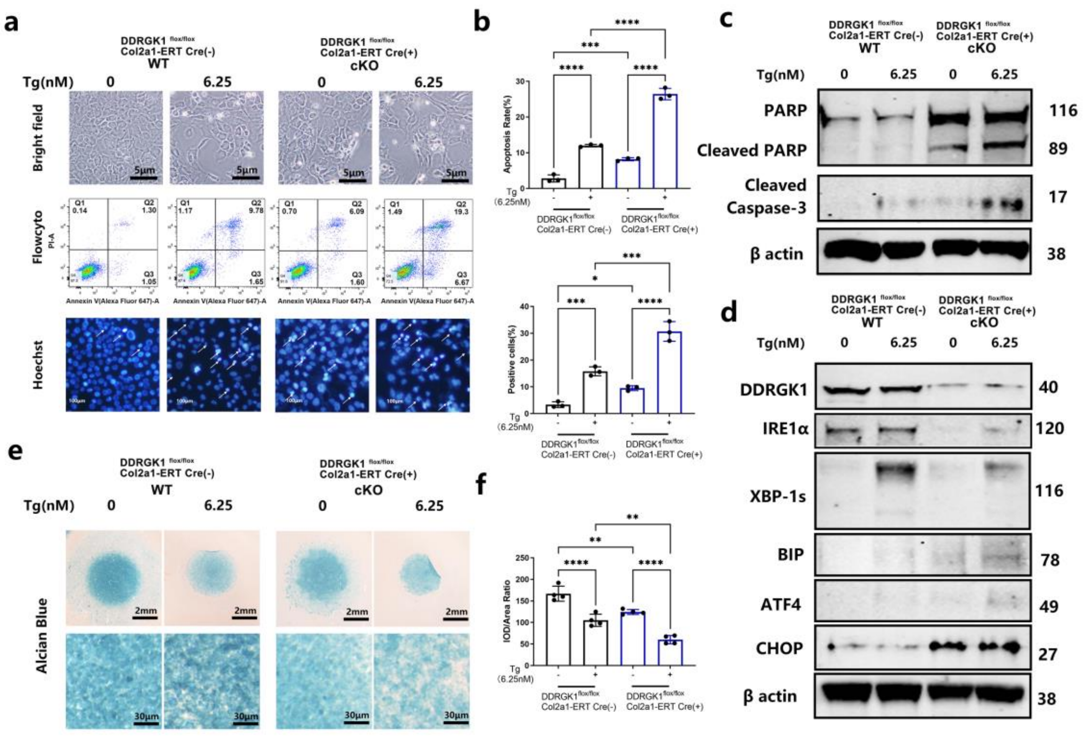

Supplementary Figure 5 | (a) Cell morphology, flow cytometry and Hoechst staining of WT and cKO primary chondrocytes induced with TMX (20μM) and treated with or without Tg (6.25 nM) for 24 h. Karyopyknosis of the cell nucleus was observed using Hoechst staining. (b) Quantification of the apoptosis rate (Q2 + Q3) based on flow cytometry, and karyopyknosis cells based on the number of cells with shrunken nuclei and the total number of cells with normal nuclei of the cells shown in panel (a). (c) Western blot analysis of cleaved and full-length PARP and cleaved Caspase 3 in primary in WT and cKO primary chondrocytes induced with TMX (20μM) and treated with or without Tg for 24 h; β-actin was the loading control. (d) Western blot analysis of DDRGK1, IRE1α, XBP-1s, BIP, ATF4 and CHOP of WT and cKO primary chondrocytes induced with TMX (20μM) and treated with or without Tg for 24 h; β-actin was the loading control. (e) Alcian blue staining of WT and cKO primary chondrocytes after 9 days of high-density culture in chondrogenesis medium induced with TMX (20μM) with or without thapsigargin (Tg) (6.25 nM). (f) Quantification of the integrated optical density /area ratio in the Alcian blue-stained cells shown in panel (e).

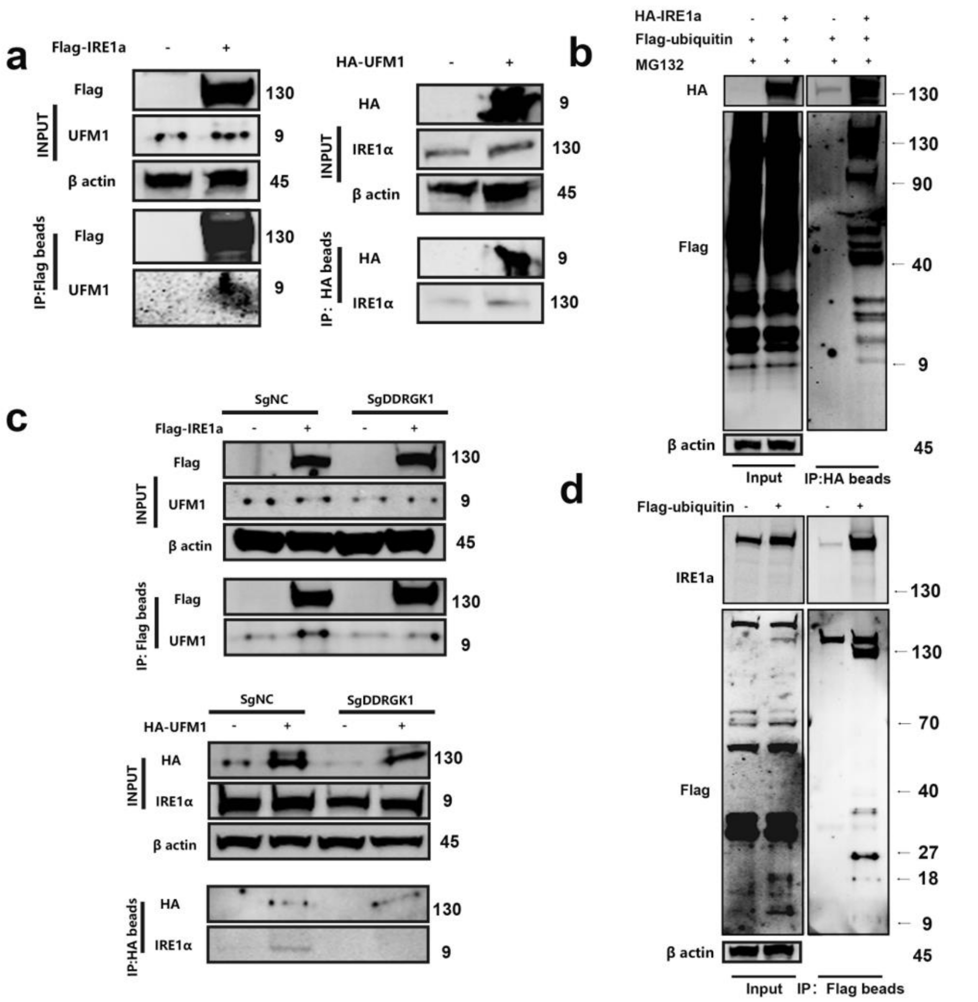

Supplementary Figure 6 | (a) Co-immunoprecipitation analysis of the possible interaction between Flag-IRE1α and UFM1, HA-UFM1 and IRE1α in 293T cells. (b) Ubiquitylation analysis of IRE1α in 293T cells treated with MG132 (10 μM) using HA-IRE1α and Flag-ubiquitin plasmids with HA-tagged beads. (c) Co-immunoprecipitation analysis of the possible interaction between Flag-IRE1α and UFM1, HA-UFM1 and IRE1α in SgNC and SgDDRGK1 ATDC5 chondrocytes. (d) Ubiquitylation analysis of intrinsic IRE1α using the Flag-Ubiquitin plasmid and Flag-tagged beads in 293T cells.

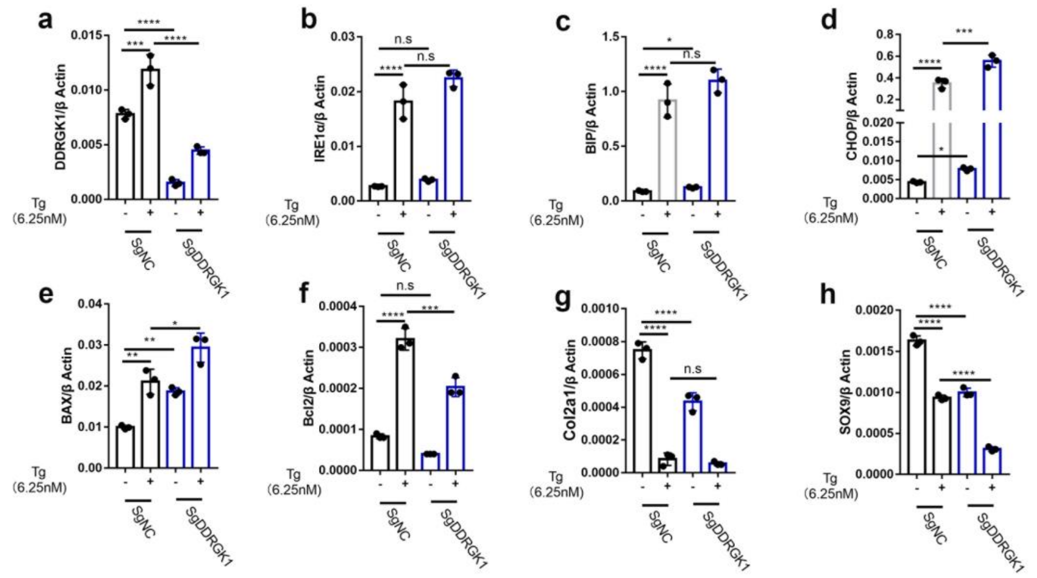

Supplementary Figure7 | (a-h) Reverse transcription-quantitative PCR analysis of the relative mRNA expression levels of DDRGK1, IRE1α, BiP, CHOP, Bax, Bcl-2, Col2a1 and SOX9 using β-actin as the internal reference in SgNC and SgDDRGK1 ATDC5 chondrocytes treated with Tg for 24 h.

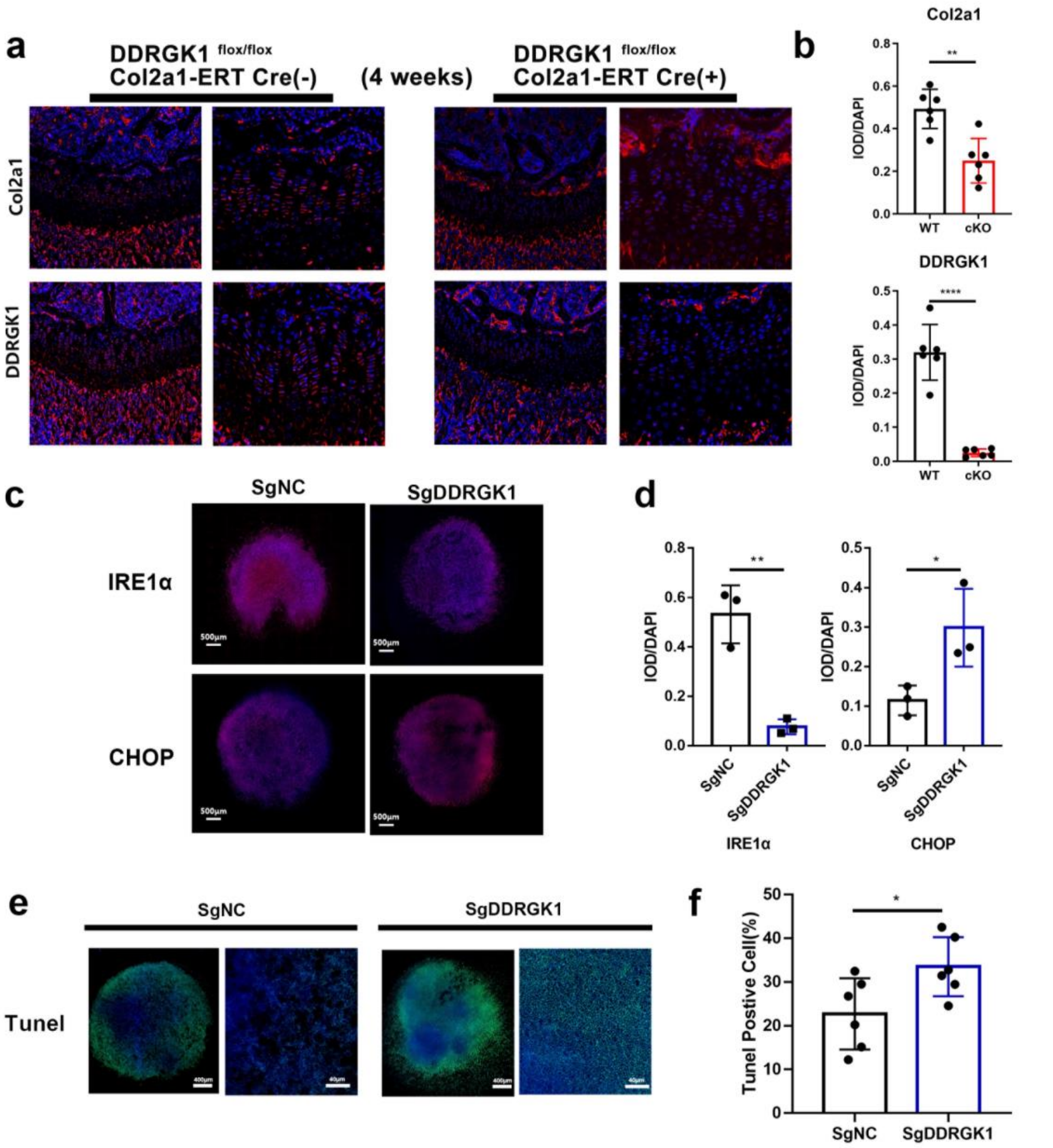

Supplementary Figure 8 | (a) Immunofluorescence analysis of Col2a1 and DDRGK1 expression in the lower limbs in WT and cKO mice shown in Figure 1A and focus on the area of growth plate. (b) Quantification of the IOD/DAPI levels of Col2a1 and DDRGK1 shown in panel (a). (c) Immunofluorescence analysis of IRE1α and CHOP expression in the pellet culture of SgNC and SgDDRGK1 ATDC5 chondrocytes 21 days after culture in chondrogenesis medium. (d) Quantification of the IOD/DAPI levels of IRE1α and CHOP expression shown in panel (c). (e) TUNEL immunofluorescence staining of the pellet culture of SgNC and SgDDRGK1 ATDC5 chondrocytes 21 days after culture in chondrogenesis medium. (f) Quantification of the percentage of TUNEL-positive cells in the pellets shown in panel (e).

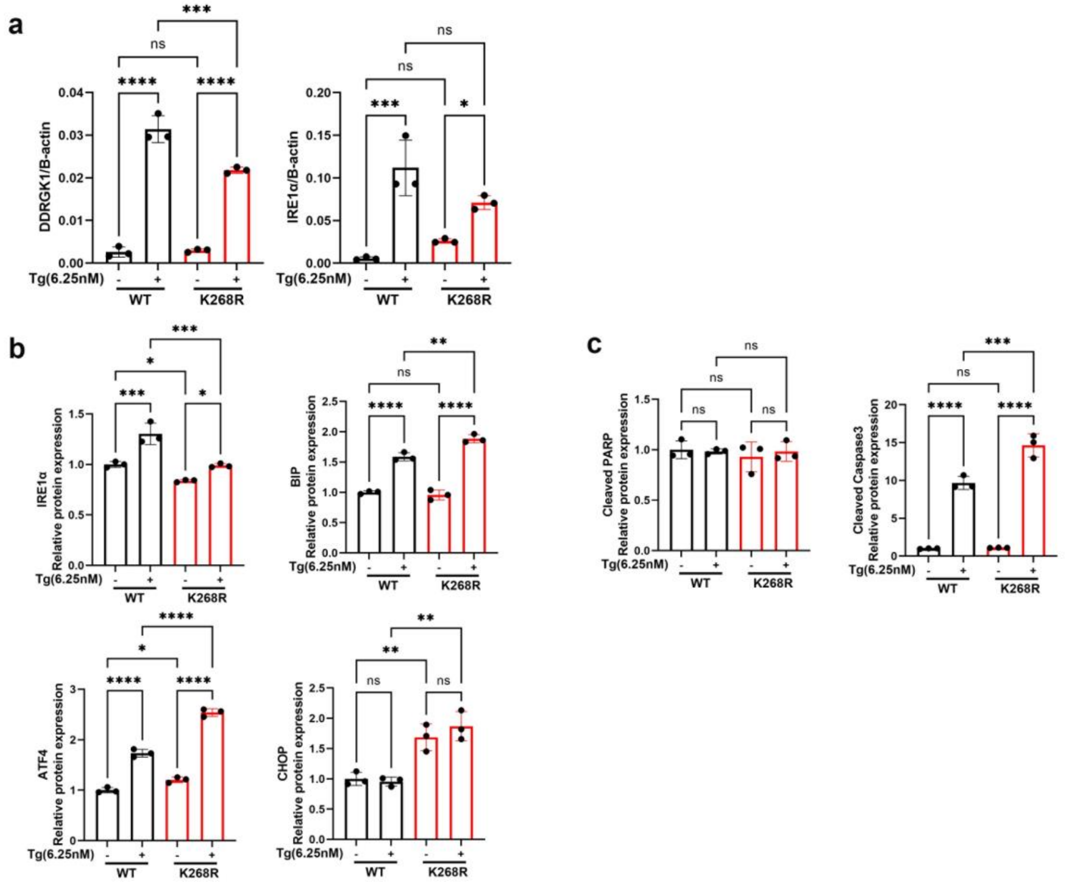

Supplementary Figure 9 | (a) Reverse transcription-quantitative PCR analysis of the relative mRNA expression levels of DDRGK1, and IRE1α using β-actin as the internal reference of primary chondrocytes treated with or without Tg for 24 h in WT and Mutant mice. (b) Quantification of the gray values of IRE1α, BIP, ATF4 and CHOP in primary chondrocytes shown in Figure 6J. (c) Quantification of the gray values of cleaved PARP and cleaved Caspase 3 in primary chondrocytes shown in Figure 6M.

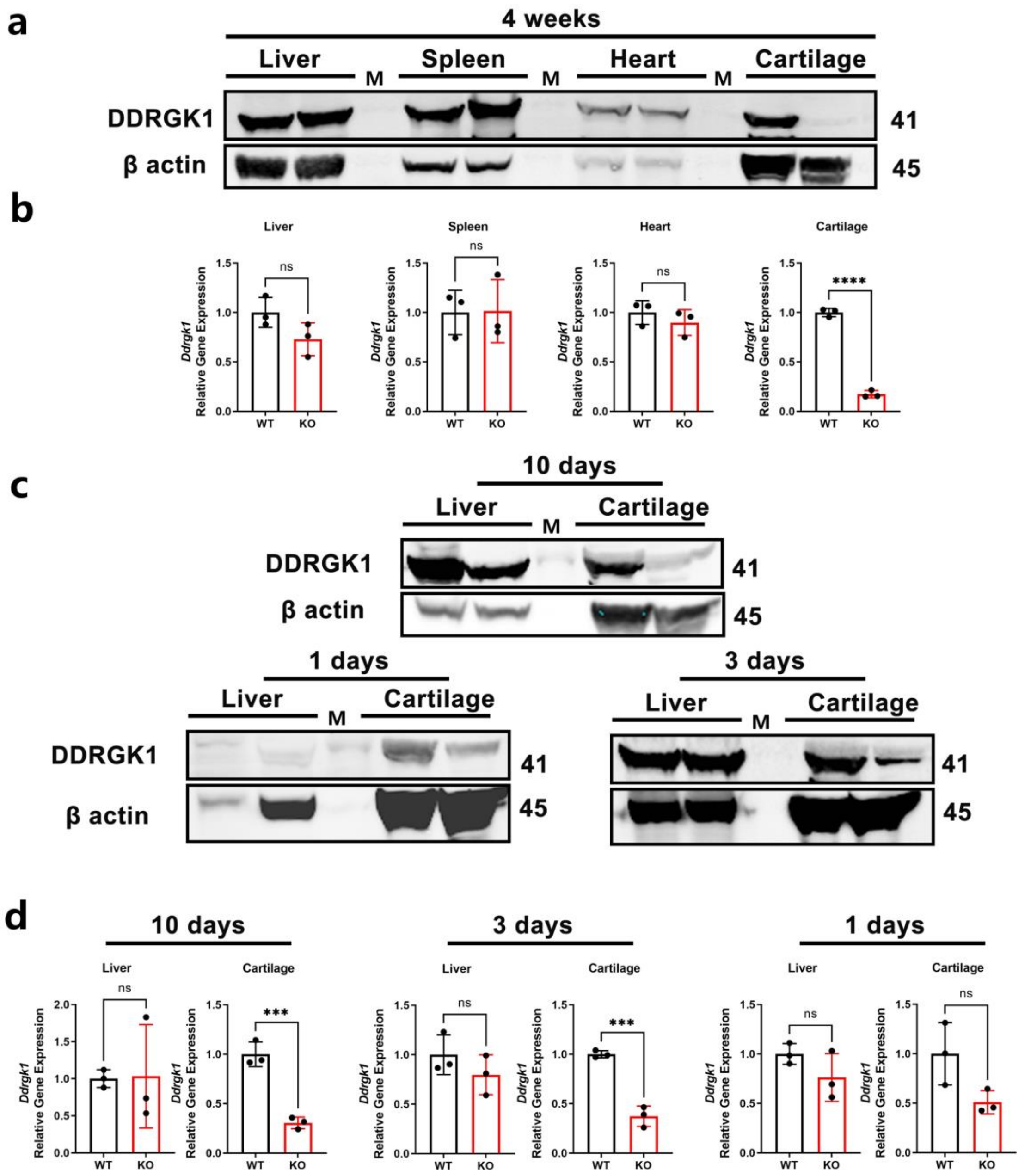

Supplementary Figure 10| (a) Western blot analysis of DDRGK1 expression using β-actin as the loading control of different organs in WT and cKO mice at 4 weeks. (b) Reverse transcription-quantitative PCR analysis of the relative mRNA expression levels of DDRGK1 expression using β-actin as the loading control of different organs in WT and cKO mice at 4 weeks. (c) Western blot analysis of DDRGK1 expression using β-actin as the loading control of heart and cartilage in WT and cKO mice at P1, P3, and P10. (d) Reverse transcription-quantitative PCR analysis of the relative mRNA expression levels of DDRGK1 expression using β-actin as the loading control of different organs in WT and cKO mice at P1, P3, and P10.

TABLE 1 | Primer sequences used for RT-qPCR.

Gene	Accession Number		5’→-3’
IRE1α	NM_023913.2.	F	CAATCGTACGGCAGTTGGAG
		R	CTCCCGGTAGTGGTGTTTCT
BiP	NM_022310.3	F	GAAAGGATGGTTAATGATGCTGAGAAG
		R	GTCTTCAATGTCCGCATCCTG
CHOP	NM_007837.4	F	CATACACCACCACACCTGAAAG
		R	CCGTTTCCTAGTTCTTCCTTGC
BAX	NM_007527.3	F	CTGGATCCAAGACCAGGGTG
		R	CCTTTCCCCTTCCCCCATTC
BCL2	NM_009741.5	F	AGCATGCGACCTCTGTTTGA
		R	GCCACACGTTTCTTGGCAAT
Col2a1	NM_001113515.2	F	AGGTGTTCGAGGAGACAGTG
		R	CAACAATGCCCCTTTGACCA
SOX9	NM_011448.4	F	TGAAGATGACCGACGAGCAG
		R	GGATGCACACGGGGAACTTA"
DDRGK1	NM_029832.2	F	GAGCACGAGGAGTACCTGAAA
		R	TCCTGAGTCCTTAGGCCCATC
